# Supplementary material for: A polygenic score method boosted by non-additive models
Source: Nat Commun. 2024 May 29;15:4433. doi: 10.1038/s41467-024-48654-x (PMC11522481; doi:10.1038/s41467-024-48654-x)
Supplement: Supplementary file 1 — Supplementary Information [file 41467_2024_48654_MOESM1_ESM.pdf]

Supplementary Methods

1 Proposed Boosting Algorithm

1.1 Polygenic score function

To define the linear polygenic score (PGS) function, assume that we are given the following input training dataset:

$$\{(\boldsymbol{x}_1, y_1), (\boldsymbol{x}_2, y_2), ..., (\boldsymbol{x}_N, y_N)\},$$

where N is the number of samples, and y_i has a Boolean value ($+1$ or -1) that expresses whether the i-th sample is diseased ($+1$) or healthy (-1). Vector $\boldsymbol{x}_i$ has the form

$$(G_{i,1}, G_{i,2}, ..., G_{i,M}, sex_i, age_i, PC_{i,1}, ...PC_{i,10})^T,$$

where M is the number of SNVs, $G \in R^{N \times M}$ is the genotypic dosage matrix, and $G_{i,j}$ is the allelic dosage, defined as the count of minor alleles, of the j-th SNV of the i-th sample ($G_{i,j} \in \{0, 1, 2\}$) after substituting missing values into the mode count. sex_i and age_i are the sex and age of the i-th sample, respectively, and $PC_{i,j}$ is the value of the j-th projected principal components of the i-th sample.

Herein, we define a polygenic score PGS for predicting the disease risk of the i-th sample $(\boldsymbol{x}_i, y_i)$ as a linear model of general functions:

$$PGS(\boldsymbol{x}_i) = \sum_{t=0}^{T-1} f_t(\boldsymbol{x}_i),$$

where $f_t(x)$ is a function from $\mathcal{X}$ to $\mathcal{R}$ ($\mathcal{X}$ is the space of all possible instances), and T is the iteration number. In typical PGS methods, each of f_t models the additive effect of a single SNV.

1.2 LogitBoost algorithm

We explain the LogitBoost algorithm [1, 2] in terms of human genetics.

LogitBoost uses real-valued functions $f_t(\boldsymbol{x}_i)$ to predict the quantitative risk of disease. We first explain how the LogitBoost algorithm works.

LogitBoost
Given: training data $\{(\boldsymbol{x}_1, y_1), (\boldsymbol{x}_2, y_2), ..., (\boldsymbol{x}_N, y_N)\}$
Initialize: $F_0(\boldsymbol{x}) = 0$
For $t = 0, ..., T-1$:

1. Compute the probability $p_{t,i}$ of sample i being a disease, the sample working response $z_{t,i}$, and the sample weight $w_{t,i}$:

$$p_{t,i} = \frac{1}{1 + \exp(-F_t(\boldsymbol{x}_i))}$$
$$z_{t,i} = \begin{cases} \frac{1}{p_{t,i}} & y_i = +1 \\ -\frac{1}{1-p_{t,i}} & y_i = -1 \end{cases}$$
$$w_{t,i} = p_{t,i}(1 - p_{t,i}).$$

2. Search SNV functions $\mathcal{H}$ for the function $f_t \in \mathcal{H}$ that minimizes

$$\sum_{i=1}^{N} w_{t,i}(f_t(\boldsymbol{x}_i) - z_{t,i})^2,$$

and select the best fit function f_t^*.
3. Update the predictor:

$$F_{t+1}(\boldsymbol{x}) = F_t(\boldsymbol{x}) + f_t^*(\boldsymbol{x}).$$

Output: $PGS(\boldsymbol{x}) = F_T(\boldsymbol{x})$

We perform T rounds of iteration to output T tests f_t. We first prepare SNV functions $\mathcal{H}$. A representative example of SNV functions $\mathcal{H}$ is the additive model of every SNV. In Step 1, we calculate the working response $z_{t,i}$ and sample weight $w_{t,i}$. In Step 2, we fit and find the SNV function to minimize the least-squares loss function. To find the best fit SNV function f_t^*, we fit every function f_t in $\mathcal{H}$ by a weighted least-squares regression to $z_{t,i}$ and select the SNV function with the minimal least-squares loss function.

1.3 LogitBoost minimizes the logistic loss function using Newton's method

Let F_t denote a tentative polygenic score function at the t-th iteration:

$$F_t(\boldsymbol{x}_i) = \sum_{\tau=0}^{t-1} f_\tau(\boldsymbol{x}_i),$$

where $f_\tau(\boldsymbol{x}_i) : \mathcal{X} \to \mathcal{R}$ is the weak learner selected in the τ-th iteration.

We consider the score function $F_t(\boldsymbol{x})$ at the t-th iteration to $F_t(\boldsymbol{x}) + f(\boldsymbol{x})$, using Newton's method, i.e., considering the quadratic approximation of the loss function.

Given a general twice differentiable loss function $l(F(\boldsymbol{x}))$, let $F_t(\boldsymbol{x})$ be the score function at the t-th iteration and consider updating it to $F_t(\boldsymbol{x}) + f(\boldsymbol{x})$. We seek a function $f(\boldsymbol{x})$ to minimize the total loss function for N samples:

$$\bar{L}_t(f(\boldsymbol{x})) = \sum_{i=1}^{N} l(F_t(\boldsymbol{x}_i) + f(\boldsymbol{x}_i)).$$

Minimizing $\bar{L}_t(f(\boldsymbol{x}))$ itself is hard, so we instead minimize the sum of the quadratic approximation of the loss function $l(F(\boldsymbol{x}))$ for N samples, which is called Newton's method.

Theorem 1 *Minimizing the sum of the quadratic approximation of the loss function $l(F(\boldsymbol{x}))$ for N samples is equivalent to minimizing the least-squares loss function $L_t(f(\boldsymbol{x}))$:*

$$L_t(f(\boldsymbol{x})) = \sum_{i=1}^{N} H_t(\boldsymbol{x}_i, y_i) \left(f(\boldsymbol{x}_i) - \frac{s_t(\boldsymbol{x}_i, y_i)}{H_t(\boldsymbol{x}_i, y_i)} \right)^2,$$

where $s_t(\boldsymbol{x}, y) = -\left.\frac{\partial l(F_t(\boldsymbol{x})+f(\boldsymbol{x}))}{\partial f(\boldsymbol{x})}\right|_{f(\boldsymbol{x})=0}$ is the negative first derivative of $l(F(\boldsymbol{x}))$ and $H_t(\boldsymbol{x}, y) = \left.\frac{\partial^2 l(F_t(\boldsymbol{x})+f(\boldsymbol{x}))}{\partial f(\boldsymbol{x})^2}\right|_{f(\boldsymbol{x})=0}$ is the second derivative of $l(F(\boldsymbol{x}))$, both evaluated at $f(\boldsymbol{x}) = 0$.

Besides, on the update, the total loss function $L_t(f(\boldsymbol{x}))$ decreases by $\Delta L_t(f(\boldsymbol{x}))$:

$$\Delta L_t(f(\boldsymbol{x})) = \frac{1}{2}\sum_{i=1}^{N} H_t(\boldsymbol{x}_i, y_i) \left(f(\boldsymbol{x}_i) - \frac{s_t(\boldsymbol{x}_i, y_i)}{H_t(\boldsymbol{x}_i, y_i)} \right)^2 - \frac{1}{2}\sum_{i=1}^{N} \frac{s_t(\boldsymbol{x}_i, y_i)^2}{H_t(\boldsymbol{x}_i, y_i)}.$$

Proof Using Newton's method, we minimize the quadratic approximation of the updated loss function $l(F_t(\boldsymbol{x})) + f(\boldsymbol{x}))$ around $F_t(\boldsymbol{x})$ for each sample:

$$\begin{aligned} l(F_t(\boldsymbol{x}) + f(\boldsymbol{x})) &\approx l(F_t(\boldsymbol{x})) + \left.\frac{\partial l(F_t(\boldsymbol{x}) + f(\boldsymbol{x}))}{\partial f(\boldsymbol{x})}\right|_{f(\boldsymbol{x})=0} f(\boldsymbol{x}) \\ &\qquad + \frac{1}{2}\left.\frac{\partial^2 l(F_t(\boldsymbol{x}) + f(\boldsymbol{x}))}{\partial f(\boldsymbol{x})^2}\right|_{f(\boldsymbol{x})=0} f(\boldsymbol{x})^2 \\ &= l(F_t(\boldsymbol{x})) - s_t(\boldsymbol{x}, y) f(\boldsymbol{x}) + \frac{1}{2} H_t(\boldsymbol{x}, y) f(\boldsymbol{x})^2. \end{aligned}$$

where $s_t(\boldsymbol{x}, y) := -\left.\frac{\partial l(F_t(\boldsymbol{x})+f(\boldsymbol{x}))}{\partial f(\boldsymbol{x})}\right|_{f(\boldsymbol{x})=0}$ and $H_t(\boldsymbol{x}, y) := \left.\frac{\partial^2 l(F_t(\boldsymbol{x})+f(\boldsymbol{x}))}{\partial f(\boldsymbol{x})^2}\right|_{f(\boldsymbol{x})=0}$ as defined above. The total loss function for N samples is given by:

$$\begin{aligned} \bar{L}_t(f(\boldsymbol{x})) &= \sum_{i=1}^{N} l(F_t(\boldsymbol{x}_i) + f(\boldsymbol{x}_i)) \\ &\approx \sum_{i=1}^{N} \left(l(F_t(\boldsymbol{x}_i)) - s_t(\boldsymbol{x}_i, y_i) f(\boldsymbol{x}_i) + \frac{1}{2} H_t(\boldsymbol{x}_i, y_i) f(\boldsymbol{x}_i)^2 \right) \end{aligned}$$

$$= \sum_{i=1}^{N} l(F_t(\boldsymbol{x}_i)) + \sum_{i=1}^{N} \left(-s_t(\boldsymbol{x}_i, y_i) f(\boldsymbol{x}_i) + \frac{1}{2} H_t(\boldsymbol{x}_i, y_i) f(\boldsymbol{x}_i)^2 \right)$$

Then, the decrease in loss function $\Delta L_t(f(\boldsymbol{x}))$ ($:= L_t(f(\boldsymbol{x})) - L_t(0)$) from $f(\boldsymbol{x}) = 0$ is

$$\begin{aligned}
\Delta \bar{L}_t(f(\boldsymbol{x})) &= \left(\sum_{i=1}^{N} l(F_t(\boldsymbol{x}_i)) + \sum_{i=1}^{N} \left(-s_t(\boldsymbol{x}_i, y_i) f(\boldsymbol{x}_i) + \frac{1}{2} H_t(\boldsymbol{x}_i, y_i) f(\boldsymbol{x}_i)^2 \right) \right) \\
&\qquad - \sum_{i=1}^{N} l(F_t(\boldsymbol{x}_i)) \\
&= \sum_{i=1}^{N} \left(-s_t(\boldsymbol{x}_i, y_i) f(\boldsymbol{x}_i) + \frac{1}{2} H_t(\boldsymbol{x}_i, y_i) f(\boldsymbol{x}_i)^2 \right) \\
&= \sum_{i=1}^{N} \left(\frac{1}{2} H_t(\boldsymbol{x}_i, y_i) \left(f(\boldsymbol{x}_i) - \frac{s_t(\boldsymbol{x}_i, y_i)}{H_t(\boldsymbol{x}_i, y_i)} \right)^2 - \frac{1}{2} \frac{s_t(\boldsymbol{x}_i, y_i)^2}{H_t(\boldsymbol{x}_i, y_i)} \right) \\
&= \frac{1}{2} \sum_{i=1}^{N} H_t(\boldsymbol{x}_i, y_i) \left(f(\boldsymbol{x}_i) - \frac{s_t(\boldsymbol{x}_i, y_i)}{H_t(\boldsymbol{x}_i, y_i)} \right)^2 - \frac{1}{2} \sum_{i=1}^{N} \frac{s_t(\boldsymbol{x}_i, y_i)^2}{H_t(\boldsymbol{x}_i, y_i)},
\end{aligned}$$

where the second term is constant for $f(\boldsymbol{x}_i)$. Thus, minimizing $\Delta \bar{L}_t(f(\boldsymbol{x}))$ is equivalent to minimizing the following least-squares loss function $L_t(f(\boldsymbol{x}))$:

$$L_t(f(\boldsymbol{x})) = \sum_{i=1}^{N} H_t(\boldsymbol{x}_i, y_i) \left(f(\boldsymbol{x}_i) - \frac{s_t(\boldsymbol{x}_i, y_i)}{H_t(\boldsymbol{x}_i, y_i)} \right)^2 .$$

□

Among a variety of loss functions, the logistic loss function is commonly adopted for two-class classification problems, including predicting case or control status. The logistic loss function $\bar{L}$ for N samples is defined as the negative log-likelihood of $\mathcal{L}$:

$$\begin{aligned}
\bar{L} &= -\ln \mathcal{L} \\
&= -\ln \left(\prod_{i:y_i=+1} p_i \prod_{i:y_i=-1} (1 - p_i) \right) \\
&= -\left(\sum_{i:y_i=+1} \ln p_i + \sum_{i:y_i=-1} \ln(1 - p_i) \right).
\end{aligned}$$

Since the probability p_i of sample i being case ($y_i = +1$) is modeled as $p_i = \frac{1}{1+\exp(-F(\boldsymbol{x}_i))}$,

$$\begin{aligned}
\bar{L} &= -\left(\sum_{i:y_i=+1} \ln p_i + \sum_{i:y_i=-1} \ln(1 - p_i) \right) \\
&= -\left(\sum_{i:y_i=+1} \ln \frac{1}{1 + \exp(-F(\boldsymbol{x}_i))} + \sum_{i:y_i=-1} \ln \frac{\exp(-F(\boldsymbol{x}_i))}{1 + \exp(-F(\boldsymbol{x}_i))} \right)
\end{aligned}$$

$$
\begin{aligned}
&= -\left(\sum_{i:y_i=+1} \ln \frac{1}{1+\exp(-F(\boldsymbol{x}_i))} + \sum_{i:y_i=-1} \ln \frac{1}{1+\exp(F(\boldsymbol{x}_i))} \right) \\
&= -\left(\sum_{i:y_i=+1} -\ln\Big(1+\exp\big(-F(\boldsymbol{x}_i)\big)\Big) + \sum_{i:y_i=-1} -\ln\Big(1+\exp\big(F(\boldsymbol{x}_i)\big)\Big) \right) \\
&= -\left(\sum_{i:y_i=+1} -\ln\Big(1+\exp\big(-y_iF(\boldsymbol{x}_i)\big)\Big) + \sum_{i:y_i=-1} -\ln\Big(1+\exp\big(-y_iF(\boldsymbol{x}_i)\big)\Big) \right) \\
&= \sum_{i=1}^{N} \ln\Big(1+\exp\big(-y_iF(\boldsymbol{x}_i)\big)\Big).
\end{aligned}
$$

From Theorem 1, minimizing the approximated logistic loss function is equivalent to minimizing the least-squares loss function. We compute the least-squares loss function for the logistic loss function at the t-th iteration according to Theorem 2.

Theorem 2 *When adopting the logistic loss function* $\Big(l(F(\boldsymbol{x})) = \ln\Big(1+\exp\big(-yF(\boldsymbol{x})\big)\Big)\Big)$, *the least-squares loss function to find minimal* $f(\boldsymbol{x})$ *at the* t*-th iteration is*

$$
L_t(f(\boldsymbol{x})) = \sum_{i=1}^{N} w_{t,i}\left(f(\boldsymbol{x}_i) - z_{t,i}\right)^2,
$$

where

$$
\begin{aligned}
w_{t,i} &= p_{t,i}(1-p_{t,i}) \\
z_{t,i} &= \begin{cases} \frac{1}{p_{t,i}} & y_i = +1 \\ -\frac{1}{1-p_{t,i}} & y_i = -1 \end{cases} \\
p_{t,i} &= \frac{1}{1+\exp(-F_t(\boldsymbol{x}_i))}.
\end{aligned}
$$

Proof We seek a function $f(\boldsymbol{x})$ to minimize the logistic loss function for N samples:

$$
\bar{L}_t(f(\boldsymbol{x})) = \sum_{i=1}^{N} l(F_t(\boldsymbol{x}_i) + f(\boldsymbol{x}_i)).
$$

From Theorem 1, the corresponding least-squares loss function is $L_t(f(\boldsymbol{x})) = \sum_{i=1}^{N}\left(H_t(\boldsymbol{x}_i, y_i)\left(f(\boldsymbol{x}_i) - \frac{s_t(\boldsymbol{x}_i,y_i)}{H_t(\boldsymbol{x}_i,y_i)}\right)^2\right)$, where $s_t(\boldsymbol{x}, y) = -\frac{\partial l(F_t(\boldsymbol{x})+f(\boldsymbol{x}))}{\partial f(\boldsymbol{x})}\Big|_{f(\boldsymbol{x})=0}$ and $H_t(\boldsymbol{x}, y) = \frac{\partial^2 l(F_t(\boldsymbol{x})+f(\boldsymbol{x}))}{\partial f(\boldsymbol{x})^2}\Big|_{f(\boldsymbol{x})=0}$.

The first derivative $\frac{\partial l(F_t(\boldsymbol{x})+f(\boldsymbol{x}))}{\partial f(\boldsymbol{x})}$ for the logistic loss function is

$$
\begin{aligned}
\frac{\partial l(F_t(\boldsymbol{x}) + f(\boldsymbol{x}))}{\partial f(\boldsymbol{x})} &= \frac{\partial}{\partial f(\boldsymbol{x})} \ln\Big(1+\exp\Big(-y\big(F_t(\boldsymbol{x}) + f(\boldsymbol{x})\big)\Big)\Big) \\
&= \frac{\exp\Big(-y\big(F_t(\boldsymbol{x}) + f(\boldsymbol{x})\big)\Big)(-y)}{1+\exp\Big(-y\big(F_t(\boldsymbol{x}) + f(\boldsymbol{x})\big)\Big)}
\end{aligned}
$$

$$= -y\left(1 - \frac{1}{1+\exp\Big(-y\big(F_t(\boldsymbol{x}) + f(\boldsymbol{x})\big)\Big)}\right).$$

Thus, $s_t(\boldsymbol{x}, y)$ is

$$\begin{aligned}
s_t(\boldsymbol{x}, y) &= -\left.\frac{\partial l(F_t(\boldsymbol{x}) + f(\boldsymbol{x}))}{\partial f(\boldsymbol{x})}\right|_{f(\boldsymbol{x})=0} \\
&= y\left(1 - \frac{1}{1+\exp(-yF_t(\boldsymbol{x}))}\right) \\
&= \begin{cases} 1 - \frac{1}{1+\exp(-F_t(\boldsymbol{x}))} = 1 - p_t(\boldsymbol{x}) & y = +1 \\ -\left(1 - \frac{1}{1+\exp(F_t(\boldsymbol{x}))}\right) = -p_t(\boldsymbol{x}) & y = -1 \end{cases} \quad \text{by } 1 - p_t(\boldsymbol{x}) = \frac{1}{1+\exp(F_t(\boldsymbol{x}))} \\
&= \hat{y} - p_t(\boldsymbol{x}),
\end{aligned}$$

where $p_t(\boldsymbol{x}) \coloneqq \frac{1}{1+\exp(-F_t(\boldsymbol{x}))}$ and

$$\hat{y} \coloneqq \begin{cases} 1 & y = +1 \\ 0 & y = -1. \end{cases}$$

The second derivative is

$$\begin{aligned}
\frac{\partial^2 l(F_t(\boldsymbol{x}) + f(\boldsymbol{x}))}{\partial f(\boldsymbol{x})^2} &= \frac{\partial}{\partial f(\boldsymbol{x})}\left(-y\left(1 - \frac{1}{1+\exp\Big(-y\big(F_t(\boldsymbol{x}) + f(\boldsymbol{x})\big)\Big)}\right)\right) \\
&= (-y)\frac{\exp\Big(-y\big(F_t(\boldsymbol{x}) + f(\boldsymbol{x})\big)\Big)(-y)}{\Big(1+\exp\Big(-y\big(F_t(\boldsymbol{x}) + f(\boldsymbol{x})\big)\Big)\Big)^2} \\
&= \frac{\exp\Big(-y\big(F_t(\boldsymbol{x}) + f(\boldsymbol{x})\big)\Big)}{1+\exp\Big(-y\big(F_t(\boldsymbol{x}) + f(\boldsymbol{x})\big)\Big)} \frac{1}{1+\exp\Big(-y\big(F_t(\boldsymbol{x}) + f(\boldsymbol{x})\big)\Big)} \\
&\qquad \text{by } y^2 = 1. \\
&= \frac{1}{1+\exp\Big(y\big(F_t(\boldsymbol{x}) + f(\boldsymbol{x})\big)\Big)} \frac{1}{1+\exp\Big(-y\big(F_t(\boldsymbol{x}) + f(\boldsymbol{x})\big)\Big)}.
\end{aligned}$$

Therefore, $H_t(\boldsymbol{x}, y)$ is

$$\begin{aligned}
H_t(\boldsymbol{x}, y) &= \left.\frac{\partial^2 l(F_t(\boldsymbol{x}) + f(\boldsymbol{x}))}{\partial f(\boldsymbol{x})^2}\right|_{f(\boldsymbol{x})=0} \\
&= \frac{1}{1+\exp(yF_t(\boldsymbol{x}))} \frac{1}{1+\exp(-yF_t(\boldsymbol{x}))} \\
&= \begin{cases} p_t(\boldsymbol{x})(1 - p_t(\boldsymbol{x})) & y = +1 \\ (1 - p_t(\boldsymbol{x}))p_t(\boldsymbol{x}) & y = -1 \end{cases} \quad \text{by } 1 - p_t(\boldsymbol{x}) = \frac{1}{1+\exp(F_t(\boldsymbol{x}))} \\
&= p_t(\boldsymbol{x})(1 - p_t(\boldsymbol{x})) \\
&= w_t(\boldsymbol{x}),
\end{aligned}$$

where $w_t(\boldsymbol{x}) \coloneqq p_t(\boldsymbol{x})(1 - p_t(\boldsymbol{x}))$.

The least-squares loss function is

$$L_t(f(\boldsymbol{x})) = \sum_{i=1}^{N} H_t(\boldsymbol{x}_i, y_i)\left(f(\boldsymbol{x}_i) - \frac{s_t(\boldsymbol{x}_i, y_i)}{H_t(\boldsymbol{x}_i, y_i)}\right)^2$$

$$= \sum_{i=1}^{N} w_t(\boldsymbol{x}_i) \left(f(\boldsymbol{x}_i) - \frac{\hat{y}_i - p_t(\boldsymbol{x}_i)}{w_t(\boldsymbol{x}_i)} \right)^2$$

$$= \sum_{i=1}^{N} w_t(\boldsymbol{x}_i) \left(f(\boldsymbol{x}_i) - z_t(\boldsymbol{x}_i, y_i) \right)^2$$

where

$$z_t(\boldsymbol{x}_i, y_i) := \frac{\hat{y}_i - p_t(\boldsymbol{x}_i)}{w_t(\boldsymbol{x}_i)} = \frac{\hat{y}_i - p_{t,i}}{p_{t,i}(1 - p_{t,i})} = \begin{cases} \frac{1}{p_{t,i}} & y_i = +1 \\ -\frac{1}{1-p_{t,i}} & y_i = -1. \end{cases}$$

Concisely by denoting $w_{t,i} = w_t(\boldsymbol{x}_i)$ and $z_{t,i} = z_t(\boldsymbol{x}_i, y_i)$,

$$L_t(f(\boldsymbol{x})) = \sum_{i=1}^{N} w_{t,i} \left(f(\boldsymbol{x}_i) - z_{t,i} \right)^2 .$$

□

Theorem 3 *The decrease in the logistic loss function $\Delta L_t(f(\boldsymbol{x}))$ at the t-th iteration is*

$$\Delta L_t(f(\boldsymbol{x})) = \frac{1}{2} \sum_{i=1}^{N} w_{t,i} \left(f(\boldsymbol{x}_i) - z_{t,i} \right)^2 - \frac{1}{2} \sum_{i=1}^{N} w_{t,i} z_{t,i}^2 .$$

Proof The decrease in logistic loss function $\Delta L_t(f(\boldsymbol{x}))$ is

$$\Delta L_t(f(\boldsymbol{x})) = \frac{1}{2} \sum_{i=1}^{N} H_t(\boldsymbol{x}_i, y_i) \left(f(\boldsymbol{x}_i) - \frac{s_t(\boldsymbol{x}_i, y_i)}{H_t(\boldsymbol{x}_i, y_i)} \right)^2 - \frac{1}{2} \sum_{i=1}^{N} \frac{s_t(\boldsymbol{x}_i, y_i)^2}{H_t(\boldsymbol{x}_i, y_i)}$$

$$= \frac{1}{2} \sum_{i=1}^{N} w_{t,i} \left(f(\boldsymbol{x}_i) - z_{t,i} \right)^2 - \frac{1}{2} \sum_{i=1}^{N} w_{t,i} z_{t,i}^2 ,$$

where, $H_t(\boldsymbol{x}_i, y_i) = w_{t,i}$ and $s_t(\boldsymbol{x}_i, y_i) = \hat{y}_i - p_{t,i} = z_{t,i} w_{t,i}$, as shown in Theorem 2. □

1.4 Non-additive GenoBoost

When we apply LogitBoost for the polygenic score, we have two types for the weak learners that represent non-additive and additive models. For both models, we can calculate analytic solutions for the parameters of each variant.

Non-additive GenoBoost assigns genotype-dependent scores $(s_{t,0}, s_{t,1}, s_{t,2})$ for allele dosage $(0, 1, 2)$, respectively, of the variant selected at the t-th iteration as

$$f_t(\boldsymbol{x}_i) = \begin{cases} s_{t,0} & G_t(\boldsymbol{x}_i) = 0 \\ s_{t,1} & G_t(\boldsymbol{x}_i) = 1 \\ s_{t,2} & G_t(\boldsymbol{x}_i) = 2. \end{cases}$$

where $G_t(\boldsymbol{x}_i)$ is defined as genotype $G_{i,j}$ when SNV j is selected at the t-th iteration, and $G_{i,j} \in \{0, 1, 2\}$ is the allelic dosage of the j-th SNV of the i-th sample.

The scores to minimize the loss function are given by the following:

$$s^*_{t,k} = \frac{U_{t,k}}{W_{t,k}}$$

where $W_{t,k} = \sum_{i:G_t(\boldsymbol{x}_i)=k} w_{t,i}$ and $U_{t,k} = \sum_{i:G_t(\boldsymbol{x}_i)=k} w_{t,i} z_{t,i}$ (for $k = 0, 1, 2$).

Among SNV candidates, we select the SNV for the t-th iteration with the minimal loss function:

$$L_t = \sum_{i=1}^{N} w_{t,i}(f_t(\boldsymbol{x}_i) - z_{t,i})^2 = \sum_{k=0}^{2} \sum_{i:G_t(\boldsymbol{x}_i)=k} w_{t,i} \left(s^*_{t,k} - z_{t,i}\right)^2 .$$

Theorem 4 *Non-additive GenoBoost minimizes the logistic loss function using Newton's method at the t-th iteration by setting parameters $s_{t,k}$ $(k = 0, 1, 2)$ to optimal values $s^*_{t,k}$:*

$$s^*_{t,k} = \frac{U_{t,k}}{W_{t,k}}. \tag{1}$$

We assume that $W_{t,k} > 0\,(k = 0, 1, 2)$, which holds when all three genotypes are present for the SNV.

Proof We calculate $f(\boldsymbol{x})$ to minimize the least-squares loss function

$$L_t(f(\boldsymbol{x})) = \sum_{i=1}^{N} w_{t,i} \left(f(\boldsymbol{x}_i) - z_{t,i}\right)^2 = \sum_{k=0}^{2} \sum_{i:G_t(\boldsymbol{x}_i)=k} w_{t,i} \left(s_{t,k} - z_{t,i}\right)^2 .$$

$s^*_{t,k}$ fulfills $\left.\frac{\partial L_t(f(\boldsymbol{x}))}{\partial s_{t,k}}\right|_{s_{t,k}=s^*_{t,k}} = 0.$

$$\begin{aligned}
\left.\frac{\partial L_t(f(\boldsymbol{x}))}{\partial s_{t,k}}\right|_{s_{t,k}=s^*_{t,k}} &= 2 \sum_{i:G_t(\boldsymbol{x}_i)=k} w_{t,i} \left(s^*_{t,k} - z_{t,i}\right) = 0 \\
s^*_{t,k} \sum_{i:G_t(\boldsymbol{x}_i)=k} w_{t,i} &- \sum_{i:G_t(\boldsymbol{x}_i)=k} w_{t,i} z_{t,i} = 0 \\
s^*_{t,k} &= \frac{\sum_{i:G_t(\boldsymbol{x}_i)=k} w_{t,i} z_{t,i}}{\sum_{i:G_t(\boldsymbol{x}_i)=k} w_{t,i}} = \frac{U_{t,k}}{W_{t,k}} \quad \text{by } W_{t,k} > 0.
\end{aligned}$$

□

We define SNV accuracy as the absolute value of the decrease in the loss function and use it as a metric to select the SNV at each iteration. High SNV accuracy indicates a great decrease in the loss function and a large association with the phenotype.

Theorem 5 *The decrease in the logistic loss function for non-additive GenoBoost at the t-th iteration is*

$$\Delta L_t(f^*(\boldsymbol{x})) = -\frac{1}{2} \sum_{k=0}^{2} \frac{U^2_{t,k}}{W_{t,k}}.$$

If $s^*_{t,k} \neq 0$ *for some* k*, the logistic loss function is guaranteed to decrease:*

$$\Delta L_t(f^*(\boldsymbol{x})) < 0.$$

Proof From Theorem 3, the decrease in the logistic loss function at the t-th iteration is given by

$$\begin{aligned}
\Delta L_t(f^*(\boldsymbol{x})) &= \frac{1}{2}\sum_{i=1}^{N} w_{t,i}\left(f^*(\boldsymbol{x}_i) - z_{t,i}\right)^2 - \frac{1}{2}\sum_{i=1}^{N} w_{t,i} z_{t,i}^2 \\
&= \frac{1}{2}\sum_{i=1}^{N}\left(w_{t,i} f^*(\boldsymbol{x}_i)^2 - 2w_{t,i} f^*(\boldsymbol{x}_i) z_{t,i} + w_{t,i} z_{t,i}^2\right) - \frac{1}{2}\sum_{i=1}^{N} w_{t,i} z_{t,i}^2 \\
&= \frac{1}{2}\sum_{i=1}^{N}\left(w_{t,i} f^*(\boldsymbol{x}_i)^2 - 2w_{t,i} f^*(\boldsymbol{x}_i) z_{t,i}\right)
\end{aligned}$$

We substitute $f^*(\boldsymbol{x})$ with $s^*_{t,k}$,

$$\begin{aligned}
\Delta L_t(f^*(\boldsymbol{x})) &= \frac{1}{2}\sum_{k=0}^{2}\sum_{i:G_t(\boldsymbol{x}_i)=k}\left(w_{t,i} s^{*2}_{t,k} - 2w_{t,i} s^*_{t,k} z_{t,i}\right) \\
&= \frac{1}{2}\sum_{k=0}^{2}\left(s^{*2}_{t,k}\sum_{i:G_t(\boldsymbol{x}_i)=k} w_{t,i} - 2s^*_{t,k}\sum_{i:G_t(\boldsymbol{x}_i)=k} w_{t,i} z_{t,i}\right) \\
&= \frac{1}{2}\sum_{k=0}^{2}\left(s^{*2}_{t,k} W_{t,k} - 2s^*_{t,k} U_{t,k}\right). \qquad (2)
\end{aligned}$$

As $s^*_{t,k} = \frac{U_{t,k}}{W_{t,k}}$,

$$\Delta L_t(f^*(\boldsymbol{x})) = \frac{1}{2}\sum_{k=0}^{2}\left(\frac{U_{t,k}^2}{W_{t,k}^2} W_{t,k} - 2\frac{U_{t,k}}{W_{t,k}} U_{t,k}\right) = -\frac{1}{2}\sum_{k=0}^{2}\frac{U_{t,k}^2}{W_{t,k}}.$$

When $s^*_{t,k} \neq 0$ for some k, $U_{t,k} \neq 0$ and $U_{t,k}^2 > 0$ hold from Supplementary Equation (1),

$$\Delta L_t(f^*(\boldsymbol{x})) < 0,$$

since $W_{t,k} > 0$ always holds. □

1.5 Additive GenoBoost

Additive GenoBoost uses two parameters (c_t, α_t) for the variant selected at the t-th iteration as

$$f_t(\boldsymbol{x}_i) = \begin{cases} c_t & G_t(\boldsymbol{x}_i) = 0 \\ c_t + \alpha_t & G_t(\boldsymbol{x}_i) = 1 \\ c_t + 2\alpha_t & G_t(\boldsymbol{x}_i) = 2. \end{cases}$$

The scores to minimize the loss function have a complicated form compared to those of non-additive GenoBoost, as shown in Theorem 6.

Theorem 6 *Additive GenoBoost minimizes the logistic loss function using Newton's method at the t-th iteration by setting parameters* (c_t, α_t) *to optimal values* (c_t^*, α_t^*) *as*

$$c_t^* = \frac{(W_{t,1} + 4W_{t,2})U_{t,0} + 2W_{t,2}U_{t,1} - W_{t,1}U_{t,2}}{W_{t,0}W_{t,1} + W_{t,1}W_{t,2} + 4W_{t,2}W_{t,0}}$$

$$\alpha_t^* = \frac{(-W_{t,1} - 2W_{t,2})U_{t,0} + (-W_{t,2} + W_{t,0})U_{t,1} + (2W_{t,0} + W_{t,1})U_{t,2}}{W_{t,0}W_{t,1} + W_{t,1}W_{t,2} + 4W_{t,2}W_{t,0}},$$

where

$$W_{t,k} = \sum_{i:G_t(\boldsymbol{x}_i)=k} w_{t,i}, \quad U_{t,k} = \sum_{i:G_t(\boldsymbol{x}_i)=k} w_{t,i}z_{t,i} \quad \text{for } k = 0, 1, 2.$$

Proof We calculate $f(\boldsymbol{x})$ under the additive model to minimize the least-squares loss function:

$$\begin{aligned} L_t(f(\boldsymbol{x})) &= \sum_{i=1}^{N} w_{t,i} \left(f(\boldsymbol{x}_i) - z_{t,i}\right)^2 \\ &= \sum_{i:G_t(\boldsymbol{x}_i)=0} w_{t,i} \left(c_t - z_{t,i}\right)^2 + \sum_{i:G_t(\boldsymbol{x}_i)=1} w_{t,i} \left((c_t + \alpha_t) - z_{t,i}\right)^2 \\ &\qquad + \sum_{i:G_t(\boldsymbol{x}_i)=2} w_{t,i} \left((c_t + 2\alpha_t) - z_{t,i}\right)^2. \end{aligned}$$

(c_t^*, α_t^*) fulfills $\left.\frac{\partial L_t(f(\boldsymbol{x}))}{\partial c_t}\right|_{(c_t,\alpha_t)=(c_t^*,\alpha_t^*)} = 0$ and $\left.\frac{\partial L_t(f(\boldsymbol{x}))}{\partial \alpha_t}\right|_{(c_t,\alpha_t)=(c_t^*,\alpha_t^*)} = 0$.

$$\begin{cases} \left.\frac{\partial L_t(f(\boldsymbol{x}))}{\partial c_t}\right|_{(c_t,\alpha_t)=(c_t^*,\alpha_t^*)} &= \sum_{i:G_t(\boldsymbol{x}_i)=0} w_{t,i} \cdot 2(c_t^* - z_{t,i}) \\ &\quad + \sum_{i:G_t(\boldsymbol{x}_i)=1} w_{t,i} \cdot 2((c_t^* + \alpha_t^*) - z_{t,i}) \\ &\quad + \sum_{i:G_t(\boldsymbol{x}_i)=2} w_{t,i} \cdot 2((c_t^* + 2\alpha_t^*) - z_{t,i}) = 0 \\ \left.\frac{\partial L_t(f(\boldsymbol{x}))}{\partial \alpha_t}\right|_{(c_t,\alpha_t)=(c_t^*,\alpha_t^*)} &= \sum_{i:G_t(\boldsymbol{x}_i)=1} w_{t,i} \cdot 2((c_t^* + \alpha_t^*) - z_{t,i}) \\ &\quad + \sum_{i:G_t(\boldsymbol{x}_i)=2} w_{t,i} \cdot 4((c_t^* + 2\alpha_t^*) - z_{t,i}) = 0. \end{cases}$$

We collect the coefficients of c_t^* and α_t^* in each equation, and transform each equation using $W_{t,k}$ and $U_{t,k}$:

$$\begin{cases} c_t^* \left(W_{t,0} + W_{t,1} + W_{t,2}\right) + \alpha_t^* \left(W_{t,1} + 2W_{t,2}\right) + \left(-U_{t,0} - U_{t,1} - U_{t,2}\right) = 0 & (3) \\ c_t^* \left(W_{t,1} + 2W_{t,2}\right) + \alpha_t^* \left(W_{t,1} + 4W_{t,2}\right) + \left(-U_{t,1} - 2U_{t,2}\right) = 0. & (4) \end{cases}$$

$\left((3) \times \left(W_{t,1} + 4W_{t,2}\right) - (4) \times \left(W_{t,1} + 2W_{t,2}\right)\right)$ and $\left((4) \times \left(W_{t,0} + W_{t,1} + W_{t,2}\right) - (3) \times \left(W_{t,1} + 2W_{t,2}\right)\right)$ yield

$$\begin{cases} c_t^* \left(W_{t,0}W_{t,1} + W_{t,1}W_{t,2} + 4W_{t,2}W_{t,0}\right) \\ \qquad - \left((W_{t,1} + 4W_{t,2})U_{t,0} + 2W_{t,2}U_{t,1} - W_{t,1}U_{t,2}\right) = 0 \\ \alpha_t^* \left(W_{t,0}W_{t,1} + W_{t,1}W_{t,2} + 4W_{t,2}W_{t,0}\right) \\ \qquad - \left((-W_{t,1} - 2W_{t,2})U_{t,0} + (-W_{t,2} + W_{t,0})U_{t,1} + (2W_{t,0} + W_{t,1})U_{t,2}\right) = 0. \end{cases}$$

c_t^* and α_t^* are given by

$$c_t^* = \frac{(W_{t,1} + 4W_{t,2})U_{t,0} + 2W_{t,2}U_{t,1} - W_{t,1}U_{t,2}}{W_{t,0}W_{t,1} + W_{t,1}W_{t,2} + 4W_{t,2}W_{t,0}}$$

$$\alpha_t^* = \frac{(-W_{t,1} - 2W_{t,2})U_{t,0} + (-W_{t,2} + W_{t,0})U_{t,1} + (2W_{t,0} + W_{t,1})U_{t,2}}{W_{t,0}W_{t,1} + W_{t,1}W_{t,2} + 4W_{t,2}W_{t,0}}.$$

□

1.6 Learning rate

We introduce two features in GenoBoost to improve the accuracy and incorporate more SNVs: the learning rate and limiting of extreme SNV scores. The original GenoBoost only takes a few highly reliable SNVs from one LD in most cases, as once an SNV is selected, the loss functions of its correlated SNVs are adjusted to be large. This behavior is expected; however, it sometimes leads to overfitting [3]. To avoid this, we introduce the learning rate. We set parameter γ ($0 < \gamma \leq 1$) as the learning rate. After selecting an SNV, the sample score is updated as

$$F_{t+1}(\boldsymbol{x}) = F_t(\boldsymbol{x}) + \gamma f_t(\boldsymbol{x}).$$

This makes the loss function decrease much less per iteration than the original and selects more SNVs from an LD block in most cases (Supplementary Fig. 17). Among several candidate values of γ, we set γ to the optimal value in the validation dataset.

1.7 Limiting extremely large absolute SNV scores

Sometimes, the SNV scores have extremely large absolute values for non-additive GenoBoost. This happens, for example, for the low-frequency variant's score $s_{t,2}$. $s_{t,2}$ is calculated only from samples with two minor alleles (Supplementary Equation (1)), and is sometimes extremely large or small due to sampling error when the number of samples with two minor alleles is small. To avoid overfitting, we limit the score $s_{t,2}$ while the estimated genetic inheritance modes stay the same.

Let $ds_{t,1} \coloneqq s_{t,1} - s_{t,0}$ and $ds_{t,2} \coloneqq s_{t,2} - s_{t,0}$ be the score of $s_{t,1}$ and $s_{t,2}$ relative to $s_{t,0}$. As illustrated in Supplementary Figure 6, if $ds_{t,1}$ or $ds_{t,2}$ has an extreme value, $s_{t,2}$ is adjusted. Sometimes, for example, for low-frequency variants, $ds_{t,2}$ could be >10 times larger than $ds_{t,1}$; these extreme SNV scores may lead to overfitting. Since the variant is the recessive mode (Fig. 1d and Supplementary Fig. 2), we limit $s_{t,2}$ so that $ds_{t,2}$ is at most $4 \times ds_{t,1}$ since this is the border of the recessive mode and additive mode. We adjust $s_{t,2}$ similarly to the overdominant and overrecessive modes. The threshold $4 \times ds_{t,1}$ is the one dividing $ds_{t,1}$ into additive mode and dominant mode at equal intervals, and other thresholds are set so that estimated additive, dominant, and recessive modes have equal range of $ds_{t,1}$ (Supplementary Fig. 2).

Supplementary Table 1 shows how to limit the SNV scores.

Condition	Adjusted $ds_{t,2}^{adj}$
$ds_{t,2} > 4ds_{t,1}$	$ds_{t,2}^{adj} = 4ds_{t,1}$
$0 < ds_{t,2} < 0.8ds_{t,1}$	$ds_{t,2}^{adj} = 0.8ds_{t,1}$
$-4ds_{t,1} < ds_{t,2} < 0$	$ds_{t,2}^{adj} = 0$
$ds_{t,2} < -4ds_{t,1}$	$ds_{t,2}^{adj} = -4ds_{t,1}$

Supplementary Table 1: Adjusted $ds_{t,2}$ after limiting the SNV scores

We tried another adjustment shown in Supplementary Table 2 as well as no adjustment, but the accuracy was lower, as shown in Supplementary Fig. 7. The threshold $2\sqrt{2}$ is the one dividing $ds_{t,1}$ additive model and dominant model geometrically.

Condition	Adjusted $ds_{t,2}$
$2\sqrt{2}ds_{t,1} < ds_{t,2}$	$ds_{t,2}^{adj} = 2\sqrt{2}ds_{t,1}$
$0 < ds_{t,2} < \sqrt{2}ds_{t,1}$	$ds_{t,2}^{adj} = \sqrt{2}ds_{t,1}$
$-2\sqrt{2}ds_{t,1} < ds_{t,2} < 0$	$ds_{t,2}^{adj} = 0$
$ds_{t,2} < -2\sqrt{2}ds_{t,1}$	$ds_{t,2}^{adj} = -2\sqrt{2}ds_{t,1}$

Supplementary Table 2: Another adjusted $ds_{t,2}$ after limiting the SNV scores

We will show that the adjustment does not change the size relationship of the decrease in the loss function of SNVs under a specific situation.

Let us assume that at the t-th iteration, recessive SNV A has SNV scores $(s_0, s_1, s_2 > 0)$ that are adjusted to $(s_0, s_1, s_2^{adj} > 0)$, respectively, so that $ds_2 = 4ds_1$, thus changing the value of the loss function from ΔL_A to ΔL_A^{adj}. As a comparison, we consider a hypothetical SNV A', whose SNV scores are (s_0, s_1, s_2^{adj}) from the beginning, and the decrease in the loss function is $\Delta L_{A'}$. We confirm that SNV A has a smaller loss function than SNV A' ($\Delta L_A < \Delta L_{A'}$) and that the ordering of L of genetic variants remains the same even after the adjustment ($\Delta L_A^{adj} < \Delta L_{A'}$) under a common condition where W_k $(k = 0, 1, 2)$ are equal for SNV A and A'.

Theorem 7 *Assume the size relationship for the SNV score of $k = 2$ for SNV A (s_2) and the adjusted score (s_2^{adj}), which is equal to the SNV score of $k = 2$ for SNV A':*

$$s_2 > s_2^{adj} > 0,$$

and assume that W_k $(k = 0, 1, 2)$ are equal for SNV A and A'.

Then, the decrease in the logistic loss function of SNV A is smaller than A':

$$\Delta L_A < \Delta L_{A'}.$$

Also, the decrease in the logistic loss function for the adjusted SNV scores of SNV A is smaller than A':

$$\Delta L_A < \Delta L_{A'},$$

therefore, the ordering of the decrease in the loss functions stays the same.

Proof We first show $U_{A,k} = U_{A',k}$ for $k = 0, 1$ and $U_{A,2} > U_{A',2} > 0$. The SNV scores of SNV A before adjustment are (s_0, s_1, s_2), which satisfy

$$s_k = \frac{U_{A,k}}{W_{A,k}} \quad (k = 0, 1, 2),$$

from Supplementary Equation (1), where $W_{A,k} = \sum_{i:x_{A,i}=k} w_{t,i}$ and $U_{A,k} = \sum_{i:x_{A,i}=k} w_{t,i} z_{t,i}$. The SNV scores of SNV A' are $(s_0, s_1, s_2^{adj} > 0)$, which satisfy

$$s_k = \frac{U_{A',k}}{W_{A',k}} \quad (k = 0, 1) \quad \text{and} \quad s_2^{adj} = \frac{U_{A',2}}{W_{A',2}} > 0.$$

where $W_{A',k} = \sum_{i:x_{A',i}=k} w_{t,i}$ and $U_{A',k} = \sum_{i:x_{A',i}=k} w_{t,i} z_{t,i}$. Here, for $k = 0, 1$, from the assumption $W_{A,k} = W_{A',k}$,

$$s_k = \frac{U_{A,k}}{W_{A,k}} = \frac{U_{A',k}}{W_{A',k}}$$
$$U_{A,k} = U_{A',k}.$$

For $k = 2$, from the assumption $s_2 > s_2^{adj} > 0$ and $W_{A,2} = W_{A',2}$,

$$s_2 = \frac{U_{A,2}}{W_{A,2}} > s_2^{adj} = \frac{U_{A',2}}{W_{A',2}} > 0$$
$$U_{A,2} > U_{A',2} > 0.$$

We next show $\Delta L_A < \Delta L_{A'}$. From Theorem 5,

$$\Delta L_A = -\frac{1}{2} \sum_{k=0}^{2} \frac{U_{A,k}^2}{W_{A,k}}$$
$$\Delta L_{A'} = -\frac{1}{2} \sum_{k=0}^{2} \frac{U_{A',k}^2}{W_{A',k}}.$$

The difference between ΔL_A and $\Delta L_{A'}$ is

$$\begin{aligned}
\Delta L_{A'} - \Delta L_A &= -\frac{1}{2} \sum_{k=0}^{2} \frac{U_{A',k}^2}{W_{A',k}} - \left(-\frac{1}{2} \sum_{k=0}^{2} \frac{U_{A,k}^2}{W_{A,k}} \right) \\
&= \frac{1}{2} \sum_{k=0}^{2} \left(\frac{U_{A,k}^2}{W_{A,k}} - \frac{U_{A',k}^2}{W_{A',k}} \right) \\
&= \frac{1}{2} \left(\frac{U_{A,2}^2}{W_{A,2}} - \frac{U_{A',2}^2}{W_{A',2}} \right) \\
&\qquad \text{by } U_{A,k} = U_{A',k} \text{ and } W_{A,k} = W_{A',k} \ (k = 0, 1) \\
&= \frac{U_{A,2}^2 - U_{A',2}^2}{2W_{A,2}} > 0 \qquad \text{by } W_{A,2} = W_{A',2} \text{ and } U_{A,2} > U_{A',2} > 0,
\end{aligned}$$

which proves $\Delta L_A < \Delta L_{A'}$.

The inequity stays as ($\Delta L_A^{adj} < \Delta L_{A'}$), when the SNV A score s_2 is adjusted to s_2^{adj}. From Supplementary Equation (2),

$$\begin{aligned}
\Delta L_A^{adj} &= \frac{1}{2} \left(\sum_{k=0}^{1} \left(s_k^2 W_{A,k} - 2 s_k U_{A,k} \right) + \left(s_2^{adj2} W_{A,2} - 2 s_2^{adj} U_{A,2} \right) \right) \\
&= \frac{1}{2} \sum_{k=0}^{1} \left(\left(\frac{U_{A,k}}{W_{A,k}} \right)^2 W_{A,k} - 2 \frac{U_{A,k}}{W_{A,k}} U_{A,k} \right) \\
&\qquad + \frac{1}{2} \left(\left(\frac{U_{A',2}}{W_{A',2}} \right)^2 W_{A,2} - 2 \frac{U_{A',2}}{W_{A',2}} U_{A,2} \right) \\
&\qquad \text{by } s_k = \frac{U_{A,k}}{W_{A,k}} \ (k = 0, 1) \text{ and } s_2^{adj} = \frac{U_{A',2}}{W_{A',2}} \\
&= -\frac{1}{2} \sum_{k=0}^{1} \frac{U_{A,k}^2}{W_{A,k}} + \frac{1}{2} \left(\frac{U_{A',2}^2}{W_{A',2}} - \frac{2 U_{A,2} U_{A',2}}{W_{A',2}} \right) \qquad \text{by } W_{A,2} = W_{A',2}.
\end{aligned}$$

The difference between ΔL_A^{adj} and $\Delta L_{A'}$ is

$$\begin{aligned}\Delta L_{A'} - \Delta L_A^{adj} &= -\frac{1}{2}\sum_{k=0}^{2}\frac{U_{A',k}^2}{W_{A',k}} - \left(-\frac{1}{2}\sum_{k=0}^{1}\frac{U_{A,k}^2}{W_{A,k}} + \frac{1}{2}\left(\frac{U_{A',2}^2}{W_{A',2}} - \frac{2U_{A,2}U_{A',2}}{W_{A',2}}\right)\right) \\ &= \frac{1}{2}\left(-\frac{U_{A',2}^2}{W_{A',2}} - \left(\frac{U_{A',2}^2}{W_{A',2}} - \frac{2U_{A,2}U_{A',2}}{W_{A',2}}\right)\right) \\ &\qquad \text{by } U_{A,k} = U_{A',k} \text{ and } W_{A,k} = W_{A',k}\ (k=0,1) \\ &= \frac{U_{A',2}}{W_{A',2}}\left(U_{A,2} - U_{A',2}\right) > 0, \qquad \text{by } U_{A,2} > U_{A',2} > 0,\end{aligned}$$

which proves $\Delta L_A^{adj} < \Delta L_{A'}$.

□

However, in practice, the ordering of L for M SNVs is not necessarily preserved if the adjustment results in a violation of the condition $s_2 > s_2^a dj > 0$ for some SNVs. We use the decrease in loss function after adjustment ΔL^{adj} for the SNV selection.

1.8 Batch screening

It is tractable to compute the loss function for all the variants and every iteration because the analytic solution does not run a regression. However, most of the variants do not change their loss function in the next iteration when they have low correlations with the selected variant. Batch screening has been proposed to avoid unnecessary computation [4, 5]. Batch screening first computes the loss function for all variants and only computes the loss function and selects the SNVs for the smallest M_{batch} for several iterations. We can reduce the computational time without compromising accuracy.

After computing the loss function for all variants, for several iterations, we only focus on variants whose loss function is larger than the threshold, which is the M_{batch}-th smallest loss function. We recompute the loss function for all variants when M_{batch} iterations have passed.

1.9 Covariates

There are several ways to incorporate quantitative and qualitative covariates. One way is to regard the covariates as weak learners themselves. This requires regression in every iteration, as there are no analytic solutions for the effect size for the quantitative variable. Another way is to use the same qualitative boosting framework by classifying the quantitative value into qualitative categories; this requires parameters for boundaries.

We decided to run logistic regression beforehand and let the predictive function serve as the initial score function of GenoBoost ($F_{cov}(\boldsymbol{x})$). This strategy is similar to GWAS and is widely accepted. However, once the effect sizes are determined, they cannot be changed.

1.10 GenoBoost algorithm

We show the GenoBoost algorithm.

Additive and non-additive GenoBoost
Given: training data $\{(\boldsymbol{x}_1, y_1), (\boldsymbol{x}_2, y_2), ..., (\boldsymbol{x}_N, y_N)\}$
User-given parameter: learning rate $0 < \gamma \leq 1$, maximum iteration T, and M_{batch}
Initialize: $t = 0$, $F_0(\boldsymbol{x}) = F_{\text{cov}}(\boldsymbol{x})$

Repeat:

1. Compute the probability of sample i being a disease $p_{t,i}$, the sample working response $z_{t,i}$, and the sample weight $w_{t,i}$:

$$p_{t,i} = \frac{1}{1 + \exp(-F_t(\boldsymbol{x}_i))}, \quad z_{t,i} = \begin{cases} \frac{1}{p_{t,i}} & (y_i = +1) \\ -\frac{1}{1-p_{t,i}} & (y_i = -1) \end{cases}, \quad w_{t,i} = p_{t,i}(1 - p_{t,i}),$$

   and set $W_{t,k} = \sum_{i:G_t(\boldsymbol{x}_i)=k} w_{t,i}$ and $U_{t,k} = \sum_{i:G_t(\boldsymbol{x}_i)=k} w_{t,i} z_{t,i}$.
2. For each SNV, compute the fitted parameters for the function under the additive model:

$$c_t^* = \frac{(W_{t,1} + 4W_{t,2})U_{t,0} + 2W_{t,2}U_{t,1} - W_{t,1}U_{t,2}}{W_{t,0}W_{t,1} + W_{t,1}W_{t,2} + 4W_{t,2}W_{t,0}}$$

$$\alpha_t^* = \frac{(-W_{t,1} - 2W_{t,2})U_{t,0} + (-W_{t,2} + W_{t,0})U_{t,1} + (2W_{t,0} + W_{t,1})U_{t,2}}{W_{t,0}W_{t,1} + W_{t,1}W_{t,2} + 4W_{t,2}W_{t,0}}.$$

   To use the non-additive model, set: $s_{t,k}^* = \frac{U_{t,k}}{W_{t,k}}$.
3. Compute the loss function: $\sum_{i=1}^{N} w_{t,i}(f_t(\boldsymbol{x}_i) - z_{t,i})^2$, where $f_t(x_i)$ is the additive (left) or non-additive (right) function defined below:

$$f_t(\boldsymbol{x}_i) = \begin{cases} c_t^* & G_t(\boldsymbol{x}_i) = 0 \\ c_t^* + \alpha_t^* & G_t(\boldsymbol{x}_i) = 1 \\ c_t^* + 2\alpha_t^* & G_t(\boldsymbol{x}_i) = 2, \end{cases} \quad f_t(\boldsymbol{x}_i) = \begin{cases} s_{t,0}^* & G_t(\boldsymbol{x}_i) = 0 \\ s_{t,1}^* & G_t(\boldsymbol{x}_i) = 1 \\ s_{t,2}^* & G_t(\boldsymbol{x}_i) = 2 \end{cases}$$

   and limit extreme SNV scores for the non-additive function.
4. Create a batch of the M_{batch} smallest SNVs.
   Initialize the batch counter $t_{batch} = 0$.
   Repeat:
   (a) Select the SNV with the smallest loss function.
   (b) Update the predictor: $F_{t+1}(\boldsymbol{x}) = F_t(\boldsymbol{x}) + \gamma f_t^*(\boldsymbol{x})$.
   (c) Increment the counter $t = t + 1$, $t_{batch} = t_{batch} + 1$
   (d) Compute the loss function according to Steps 1, 2, and 3 for batch SNVs.
   (e) If $t_{batch} = M_{batch}$ or $t = T$, exit the inner loop of Step 4.
5. If $t = T$, exit the loop.

Output: $F_T(\boldsymbol{x})$

1.11 Computational complexity

The order of the computational time of GenoBoost is $O(NMT_{batch} + NM_{batch}T)$, where N is the number of samples, M is the number of candidate SNVs, M_{batch} is the number of SNVs in the batch, T is the number of iterations, and T_{batch} is the number of iterations to compute the loss function for all M variants.

We devised an implementation to improve speed and memory consumption using the compressed 2-bit form for genotype [6], single instruction, multiple data (SIMD) instructions, and multithreading programming. GenoBoost is capable of processing millions of SNVs in a reasonable time.

1.12 Estimation of genetic modes

We can estimate genetic inheritance modes from the scores of the selected SNVs. We obtain a score of the heterozygote relative to that of the minor allele homozygotes, defined as $s_1 - s_0$ and denoted by ds_1, and that of the major allele homozygotes, defined as $s_2 - s_0$ and denoted by ds_2. We obtain the ratio of the two relative scores $rs = \frac{ds_1}{ds_2}$. From these scores, we estimate the genetic inheritance mode of a selected SNV as being overdominant, dominant, additive, recessive, or overrecessive, as shown in Supplementary Table 3 and Figure 1d. We first classify variants into the dominant, additive, recessive, and heterozygotes-only modes. We then classify the heterozygotes-only modes into the overdominant and overrecessive modes by the sign of ds_1.

Inferred genetic inheritance mode	Condition
Overdominant	$(rs < -\frac{1}{4}$ or $\frac{5}{4} < rs\ )$ and $ds_1 > 0$
Dominant	$\frac{3}{4} < rs < \frac{5}{4}$
Additive	$\frac{1}{4} < rs < \frac{3}{4}$
Recessive	$-\frac{1}{4} < rs < \frac{1}{4}$
Overrecessive	$(rs < -\frac{1}{4}$ or $\frac{5}{4} < rs\ )$ and $ds_1 < 0$

Supplementary Table 3: Genetic inheritance mode classification

2 Data preparation

2.1 Phenotypic definition

We set case/control labels for samples based on self-reported medical conditions(Data Field 20001/20002) and health record information, including International Classification of Diseases (ICD-9, ICD-10) codes and OPCS Classification of Interventions and Procedures (OPCS-4) codes. Supplementary Table 4 shows the definition of phenotypes [7–12].

For cancers, including breast cancer and colorectal cancer, national cancer registries were also included [10, 12]. Gout also includes patients taking allopurinol or sulphinpyrazone therapy, excluding those who also had lymphoma or leukemia [8].

2.2 Non-European samples

To investigate the portability of polygenic scores, we calculated the accuracy of the polygenic scores of African, South Asian, and East Asian samples. We define African, South Asian, and East Asian samples using genotype principal components (PCs, defined in Data Field 22009) and the self-reported ancestry (Data Field 21000) as follows; African: $260 \leq$ PC1, $50 \leq$ PC2, and not self-identified as any of Asian, White, Mixed, or Other population groups; South Asian: $40 \leq$ PC1 ≤ 120, $-170 \leq$ PC2 ≤ -80, and not self-identified as any of Black, White, Mixed, or Other population groups; and East Asian $130 \leq$ PC1 ≤ 170, PC2 ≤ -230, and not self-identified as any of Black, White, Mixed, or Other population groups [13]. For quality control, we further excluded samples registered as putative sex chromosome aneuploidy or outliers for heterozygosity or missing rate. The African sample sizes were 6,487 and 3,704 for females, the South Asian sample sizes were 7,952 and 3,604 for females, and the East Asian sample sizes were 1,770 and 1,138 for females. Supplementary Table 5 shows case/control sample information for non-European samples.

We randomly split each of them into 20% validation and 80% test datasets. We used the validation dataset to estimate the effects of covariates and the test dataset to evaluate the predictive performance of PGS models.

2.3 PGS models without genetic variants on Chromosome 6

To investigate whether non-additive GenoBoost captures non-additive effects of other regions than the MHC region, we excluded chromosome 6 from the polygenic score function and computed the accuracy for rheumatoid arthritis, psoriasis, gout, inflammatory bowel disease, and asthma. We also excluded chromosome 6 from the training dataset and trained for GenoBoost and LDpred for rheumatoid arthritis and psoriasis.

Phenotype	self-reported (Data Field: code)	ICD , OPCS-4 (ICD,OPCS: code)	Others	Reference
Rheumatoid arthritis	DF20002: 1464	ICD10: M05,M06	-	[8]
Psoriasis	DF20002: 1453	ICD9: 691.0,696.1<br>ICD10: L40	-	[9]
Gout	DF20002: 1466	ICD10: M10	DF20003: 1140875408, 1140875496<br>but not in ICD10: C81-C96	[8]
Inflammatory bowel disease	DF20002: 1461	ICD9: 555.*<br>ICD10: I48<br>OPCS4: K57.1,K62.1-4	-	[10]
Asthma	DF20002: 1111	ICD9: 493.*<br>ICD10: J45	-	UK Biobank Resource 460
All-cause dementia	DF20002: 1263	ICD9: 290.2-4,291.2,294.1, 31.0-2,331.5	-	[11]
Alzheimer's disease	-	ICD9: 331.0<br>ICD10: F00,G30	-	[11]
Atrial fibrillation	DF20002: 1471,1483	ICD9: 427.3<br>ICD10: I48<br>OPCS4: K57.1,K62.1-4	-	[10]
Breast cancer	DF20001: 1002	ICD9: 174,174.9<br>ICD10: C50	Include national cancer registry	[10]
Colorectal cancer	DF20001: 1020,1022	ICD10: C18,C19,C20	Include national cancer registry	[12]
Coronary artery disease	DF20002: 1075	ICD9: 410.*,411.0,412.*,429.79<br>ICD10: I21,I22,I23,I24.1,I25.2<br>OPCS4: K40.1-4,K41.1-4,K45.1-5 K49.1-2,K49.8-9,K50.2, K75.1-4,K75.8-9	-	[10]
Type 2 diabetes	DF20002: 1223	ICD10: E11	-	[10]

Supplementary Table 4: Phenotype codes in UK Biobank

Phenotype	case/control sample size			case prevalence [%]			
	African	South Asian	East Asian	African	South Asian	East Asian	white British
Rheumatoid arthritis	159 / 6328	254 / 7698	22 / 1748	2.3	3.2	1.2	2.4
Psoriasis	25 / 6462	137 / 7815	10 / 1760	0.4	1.7	0.6	2.0
Gout	150 / 6337	243 / 7709	47 / 1723	2.2	3.1	2.7	2.8
Inflammatory bowel disease	37 / 6450	124 / 7828	7 / 1763	0.5	1.6	0.4	1.1
Asthma	940 / 5547	1317 / 6635	192 / 1578	13.7	16.6	10.8	14.3
All-cause dementia	101 / 6386	93 / 7859	6 / 1764	1.5	1.2	0.3	1.5
Alzheimer's disease	42 / 6445	35 / 7917	3 / 1767	0.6	0.4	0.2	0.6
Atrial fibrillation	210 / 6277	342 / 7610	44 / 1726	3.1	4.3	2.5	7.7
Breast cancer	161 / 3543	200 / 3404	64 / 1074	4.3	5.5	5.6	7.6
Colorectal cancer	76 / 6411	65 / 7887	21 / 1749	1.1	0.8	1.2	2.0
Coronary artery disease	220 / 6267	989 / 6963	46 / 1724	3.2	12.4	2.6	6.9
Type 2 diabetes	1511 / 5336	2074 / 5878	149 / 1621	22.1	26.1	8.4	7.6

Supplementary Table 5: The panel of twelve disease outcomes for non-European samples in UK Biobank analyzed in the study.

2.4 Simulation Study

To understand the situation where additive and non-additive GenoBoost had higher accuracy than other methods, we simulated polygenic phenotypes varying additive and non-additive heritability (h^2_{add}, h^2_{dom}) and the number of causal SNVs (M_{causal}). We used the UK Biobank genotype of unrelated white British samples. The overall heritability (h^2), which is the sum of additive and non-additive heritability, was set to 0.05 and 0.1, the numbers of causal SNVs were 100 and 1000, the proportions of dominance heritabilities were 0% and 20% (Supplementary Table 6). Ten simulation datasets for each parameter were generated according to the procedure below.

h^2	h^2_{add}	h^2_{dom}	M_{causal}
0.05	0.05	0.0	100 / 1000
0.05	0.04	0.01	100 / 1000
0.1	0.1	0.0	100 / 1000
0.1	0.08	0.02	100 / 1000

Supplementary Table 6: Simulation parameter

Generation of Simulation Dataset

User-given parameters: h^2_{add}, h^2_{dom}, M_{causal}, case prevalence p

1. Generate a set of causal SNVs.

$$S_{causal} \leftarrow M_{causal} \text{ SNVs that are randomly selected}$$

2. Generate additive and dominance effect sizes.
   For the j-th SNV,

$$\beta_j^{add} = 0 \qquad j \notin S_{causal}$$
$$\beta_j^{add} \sim N\left(0, \frac{h^2_{add}}{M_{causal}}\right) \quad j \in S_{causal},$$

   and

$$\beta_j^{dom} = 0 \qquad j \notin S_{causal}$$
$$\beta_j^{dom} \sim N\left(0, \frac{h^2_{dom}}{M_{causal}}\right) \quad j \in S_{causal},$$

   where $N(\cdot,\cdot)$ is normal distribution.
3. Calculate the dosage scores for each SNV.

$$\begin{bmatrix} s_{j,0} \\ s_{j,1} \\ s_{j,2} \end{bmatrix} = \begin{bmatrix} -\beta_j^{dom} \frac{f_j}{1-f_j} \\ \frac{\beta_j^{add}}{\sqrt{2f_j(1-f_j)}} + \beta_j^{dom} \\ \frac{2\beta_j^{add}}{\sqrt{2f_j(1-f_j)}} - \beta_j^{dom} \frac{1-f_j}{f_j} \end{bmatrix},$$

   where f_j is the minor allele frequency of the j-th SNV.
4. Calculate liability for the i-th sample L_i.

$$\epsilon_i \sim N(0, 1 - h^2_{add} - h^2_{dom})$$

$$L_i = \epsilon_i + \sum_{j \in S_{causal}} \begin{cases} s_{j,0} & G_{i,j} = 0 \\ s_{j,1} & G_{i,j} = 1 \\ s_{j,2} & G_{i,j} = 2. \end{cases}$$

5. Define the disease status of the i-th sample y_i; namely, $y_i = 1$ if L_i is in top p proportion and $y_i = 0$ otherwise.

3 Previously Published Polygenic Score Methods

To adjust the effects of covariates, we input both covariates and genotypes in the snpnet program for snpnet and snpboost. For lassosum, LDpred, PRS-CS, SBayesR, and C+T, we constructed PGS scores from summary statistics, which adjusted covariate effects. We applied five-fold cross-validation and optimized hyperparameters via grid search based on covariate-adjusted pseudo-R^2 in the validation dataset, following the manner of GenoBoost.

3.1 Snpnet

Snpnet [4] is an algorithm to apply Batch Screening Iterative Lasso algorithm using individual-level data. LASSO's loss function is the sum of the logistic loss function and a regularization term and differs from GenoBoost's. snpnet has one parameter, the regularization factor, which determines the number of SNVs. We used the default value for the regularization factors of snpnet, which searches for 100 values.

3.2 Snpboost

Snpboost [5] is an iterative algorithm to minimize the loss function using the boosting technique using individual-level data. Snpboost runs logistic regression at every iteration and selects the SNV with the smallest loss function. Snpboost uses a batch screening iterative method similar to snpnet. Unlike GenoBoost, snpboost cannot exploit the non-additive model and uses regression to minimize loss function in every iteration.

3.3 Lassosum

We also used lassosum (version 0.4.5) [14], a summary statistics-based LASSO. Lassosum inputs summary statistics and a reference panel for the LD matrix. The regularization factors for lassosum were 0.0005, 0.001, 0.005, 0.01, 0.0125, 0.015, 0.175, 0.02, 0.025, 0.03, 0.04, 0.05, and 0.1. We used 489 European samples from 1,000 genomes as a reference panel.

3.4 LDpred

LDpred (version 1.0.11) [15] estimates effect size as posterior mean effect assuming a non-infinitesimal model with a reference panel. Unlike C+T, LDpred can increase accuracy by capturing effects in LD regions surrounding the most associated SNVs. Setting the prior distribution can be regarded as applying regularization. LDpred has several parameters, but we only vary the fraction of causal markers here. The fractions of causal markers were 1.0, 0.1, 0.01, and 0.001. We set the radius of the LD block as 500. We used 489 European samples from 1,000 genomes as a reference panel.

3.5 PRS-CS

PRS-CS [16] is similar to LDpred but exploits the continuous priors on the effect sizes, which is favorable both theoretically and computationally. We used PRS-CS-auto,

which estimates parameter ϕ from the summary statistics. We used 489 European samples from 1,000 genomes as a reference panel.

3.6 SBayesR

SBayesR (version 2.02) [17] estimates effect sizes using finite mixture models. SBayesR does not require hyperparameters. We first tried to run SBayesR, but the convergence error was raised for several phenotypes. We used a p-value threshold for SNVs of 0.4 following the manual. This corrected the error in most phenotypes; however, the error remained for psoriasis and Alzheimer's disease and for four out of five cross-validations of gout. We used 489 European samples from 1,000 genomes as a reference panel.

3.7 C+T

C+T [18] repeats clumping, selecting the smallest p-value SNV, and thresholding, excluding SNVs correlated with selected SNVs. C+T has two parameters: the p-value and the correlation thresholds. The p-values were 0.01, 0.03, 0.1, and 0.3, and the correlations were 0.1, 0.3, 0.5, 0.7, and 0.9. We also ran C+T using the number of SNVs instead of the p-value to extract the smaller number of SNVs. The numbers were 5, every 10 from 10 to 150, every 100 from 200 to 1,000, and every 1,000 from 2,000 to 10,000. The maximum number of 10,000 is sufficient, as the numbers of SNVs used with the p-value of 0.01 were less than or close to 10,000. We used the training dataset of UK Biobank as a reference panel.

4 Non-additive snpnet

It is possible to create a non-additive version of LASSO by modifying the input genotype for snpnet. We prepare dummy non-additive genotype matrix $D \in R^{N \times M}$ created from genotypic dosage matrix $G \in R^{N \times M}$. $D_{i,j} \in \{0, 1\}$ for j-th SNV of the i-th sample is defined as $D_{i,j} = 1$ if $G_{i,j} = 1$ and $D_{i,j} = 0$ if $G_{i,j} = 0$ or 2. By inputting $[G, D]$ instead of G, snpnet outputs a non-additive polygenic function $G\beta + D\gamma$.

Supplementary Notes

Supplementary Note 1. PGS models without genetic variants on Chromosome 6

Non-additive GenoBoost had higher accuracy than other methods in rheumatoid arthritis and psoriasis, where heritability enriches in the MHC region. To investigate whether non-additive GenoBoost successfully captures non-additive effects of the region, we computed the accuracy of PGS when trained with the whole genotype and excluding chromosome 6 in the test dataset. GenoBoost tied with some other methods, which suggests that GenoBoost successfully captured non-additive effects of the MHC region, but GenoBoost still ranked first for three out of five phenotypes (Supplementary Fig. 5a). We also excluded chromosome 6 from the training dataset, and obtained results similar to excluding chromosome 6 in the test dataset (Supplementary Fig. 5b).

Supplementary Note 2. Simulation

We compared the predictive accuracy of additive and non-additive GenoBoost and LDpred on the simulation dataset defined in the Supplementary Methods. As shown in Supplementary Fig. 10, GenoBoost had higher accuracy under all simulation parameters except for one parameter setting ($h^2 = 0.05$, $h_d^2 = 0.01$, and $M_{causal} = 1000$). Non-additive GenoBoost performed better accuracy when there was dominance heritability with a large overall heritability ($h^2 = 0.1$) and tied with additive GenoBoost otherwise.

Supplementary Note 3. Non-additive snpnet

We implemented a non-additive version of snpnet [LASSO] that is described in Supplementary Methods. The non-additive snpnet improved the accuracy for psoriasis but did not outperform GenoBoost (Supplementary Fig. 11).

Supplementary Note 4. Predictive ability on African, South Asian, and East Asian samples

To investigate the portability of polygenic scores, we evaluated the covariate-adjusted pseudo-R^2 of PGS models on African, South Asian, and East Asian samples in UKB. We first fit two regression models, one with covariate terms alone and another with covariate terms and PGS score (the 'full' model), using individuals in the validation set. We subsequently evaluated the likelihood of each model using the test set individuals and reported the covariate-adjusted pseudo-R^2.

As shown in Supplementary Fig. 12, the accuracy for those populations was lower than for white British samples for most methods and phenotypes.

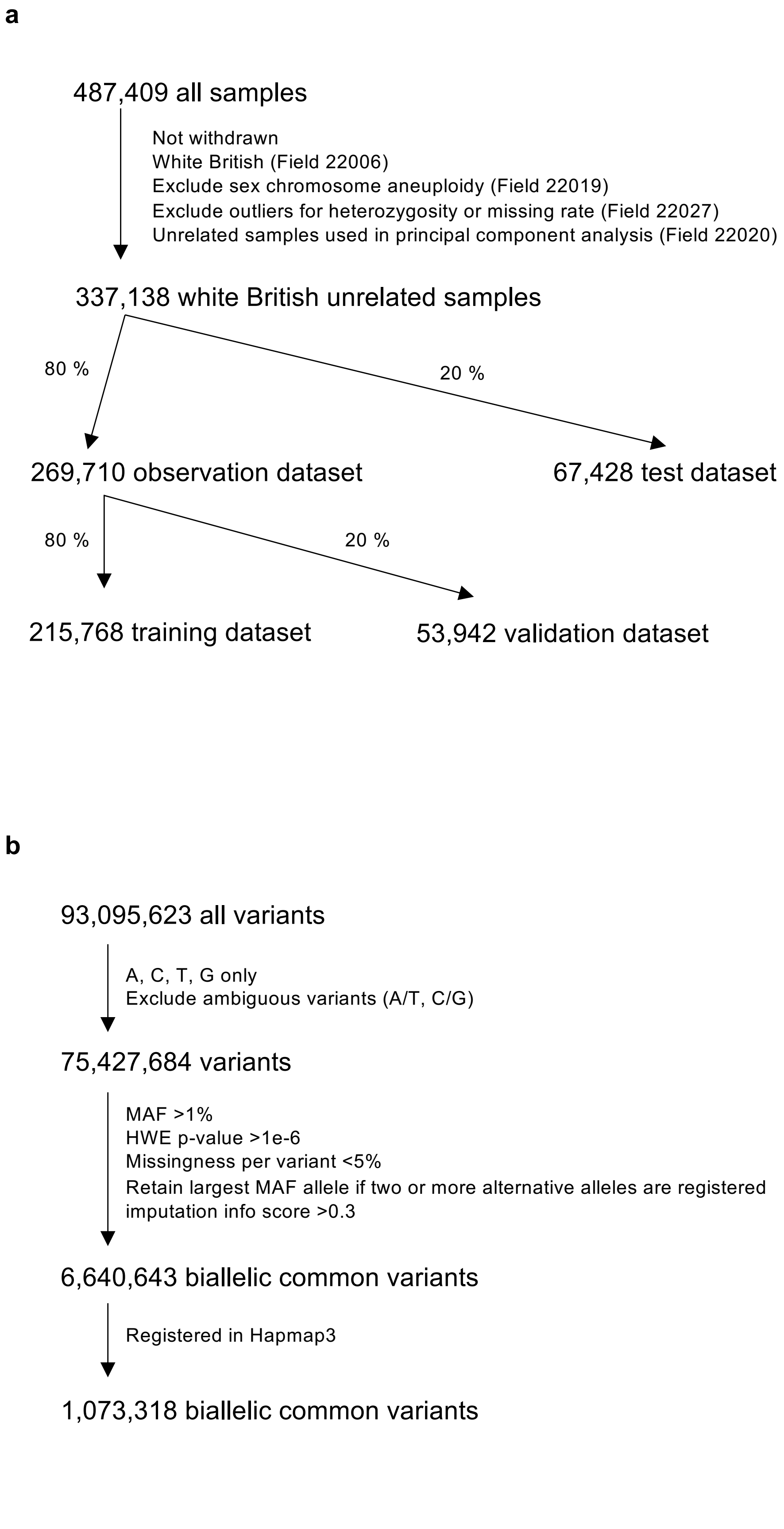

Supplementary Figure 1. **Sample and single nucleotide variant quality control (QC) summary.** Flowchart to show sample and SNV QC. After the QC, 337,138 white British unrelated samples and 1,073,318 variants remained. The training sample size was 215,768 samples.

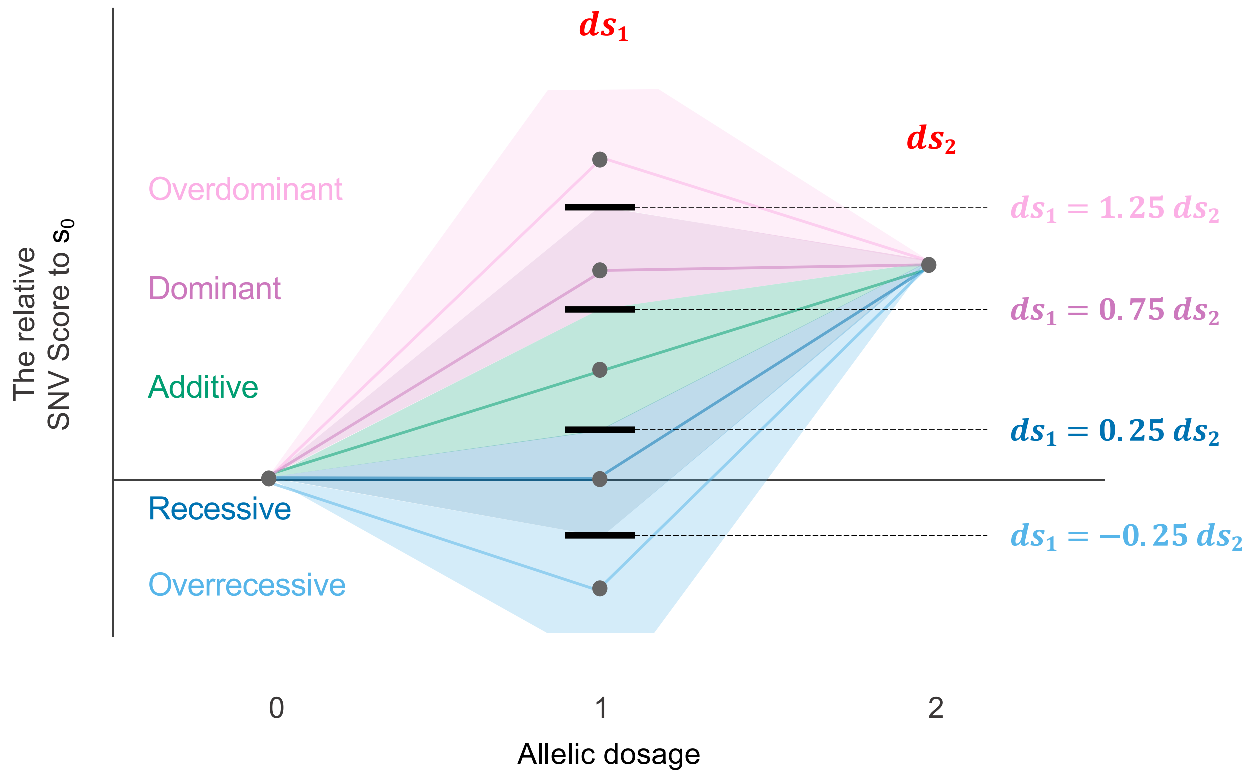

Supplementary Figure 2. **Inference of the underlying genetic inheritance mode from genotype-dependent scores.** The genetic inheritance mode is inferred using the relative SNV scores $ds_1 = s_1 - s_0$ and $ds_2 = s_2 - s_0$. The threshold linearly splits ds_1 into five inheritance modes.

a

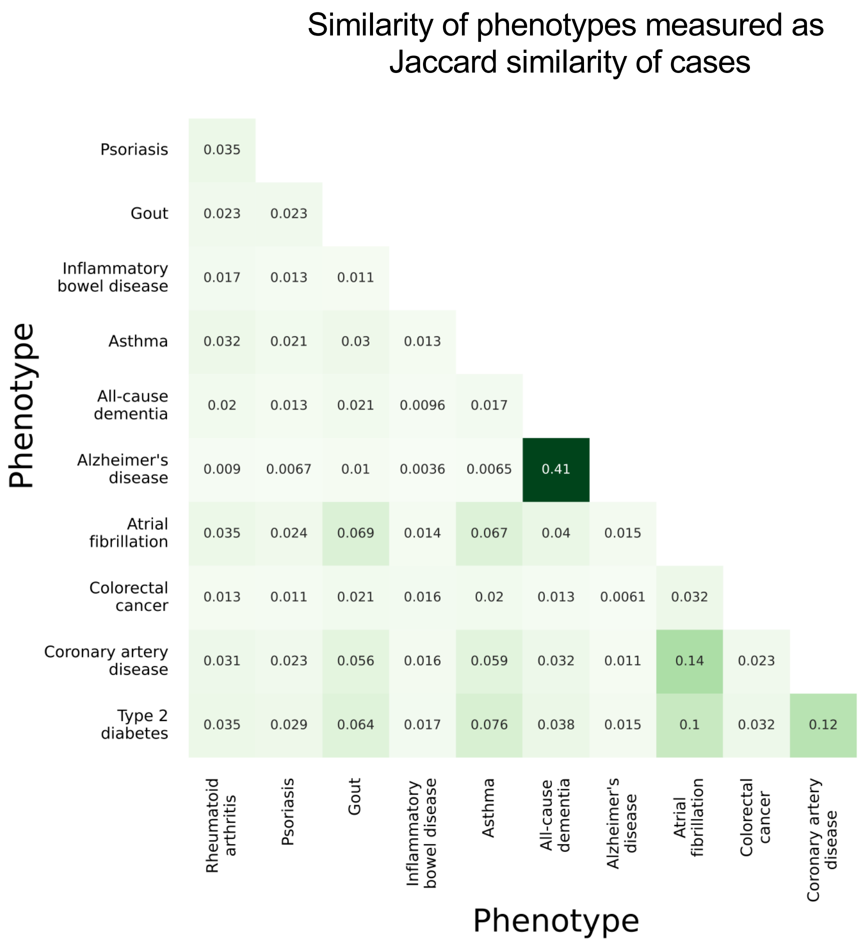

b

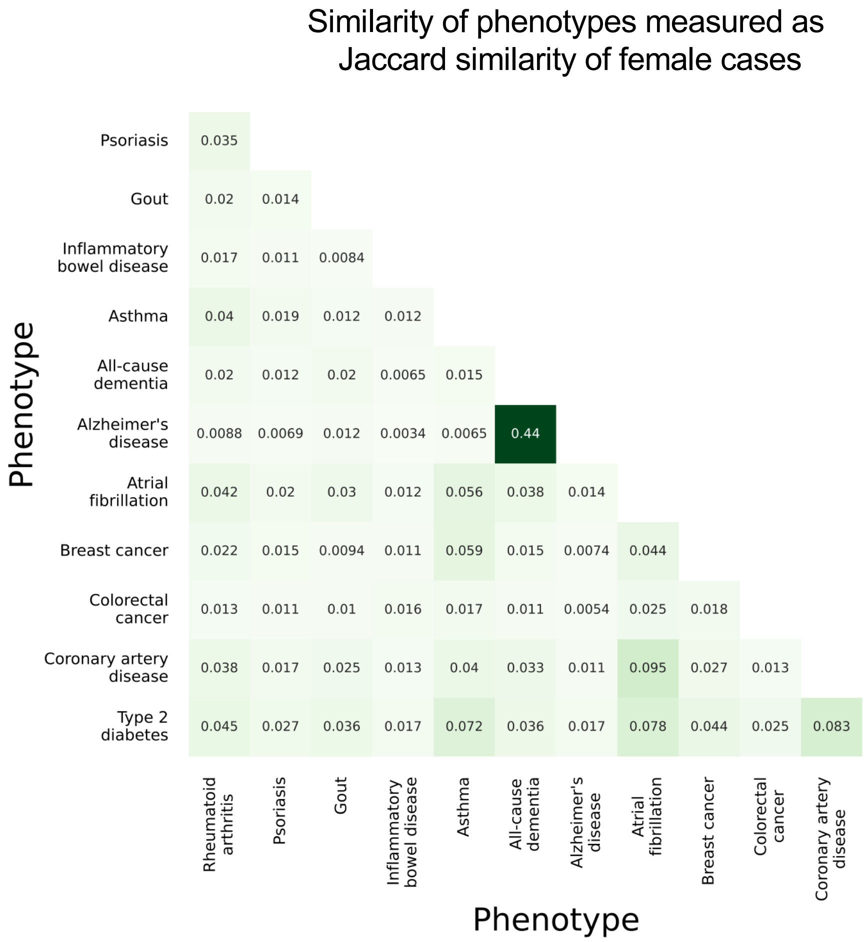

Supplementary Figure 3. **Jaccard similarity index between phenotypes for both sexes (a) and female only (b).**

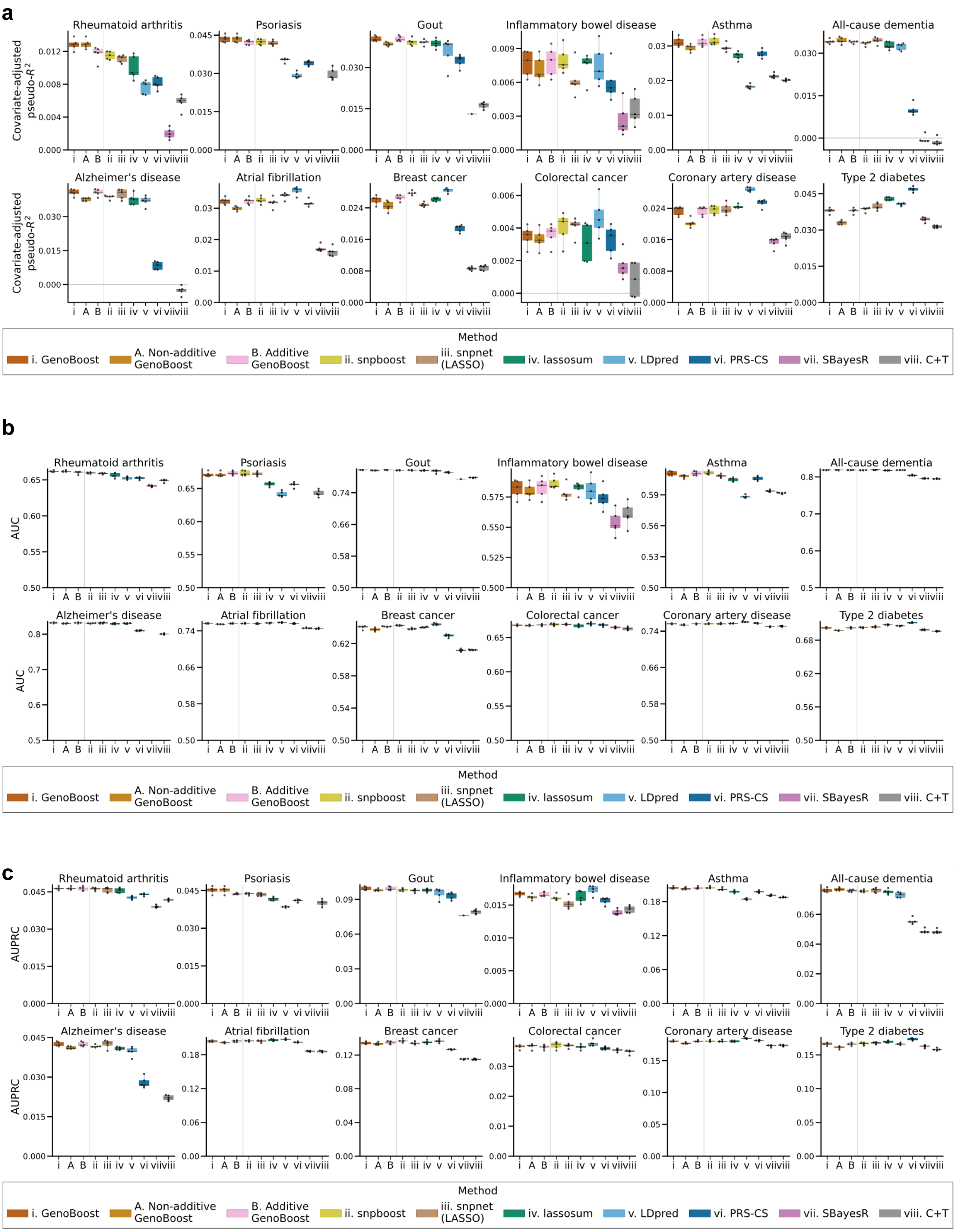

Supplementary Figure 4. **Benchmarking GenoBoost against seven commonly used methods across twelve diseases in UK Biobank. a-c** Predictive performance measured by covariate-adjusted pseudo-R^2 (**a**), area under the curve (AUC) (**b**) and area under the PR curve (AUPRC) (**c**) of PGS models across GenoBoost (i, A, B) and seven other methods (ii-viii). On each box, the center line is the median, the top and bottom of the box are the second and fourth values (Q3 and Q1), and the upper and lower whiskers are shown if the first and the fifth are in Q3 + 1.5 IQR and Q1 – 1.5 IQR, respectively, where IQR = Q3 – Q1. Source data are provided as a Source Data file.

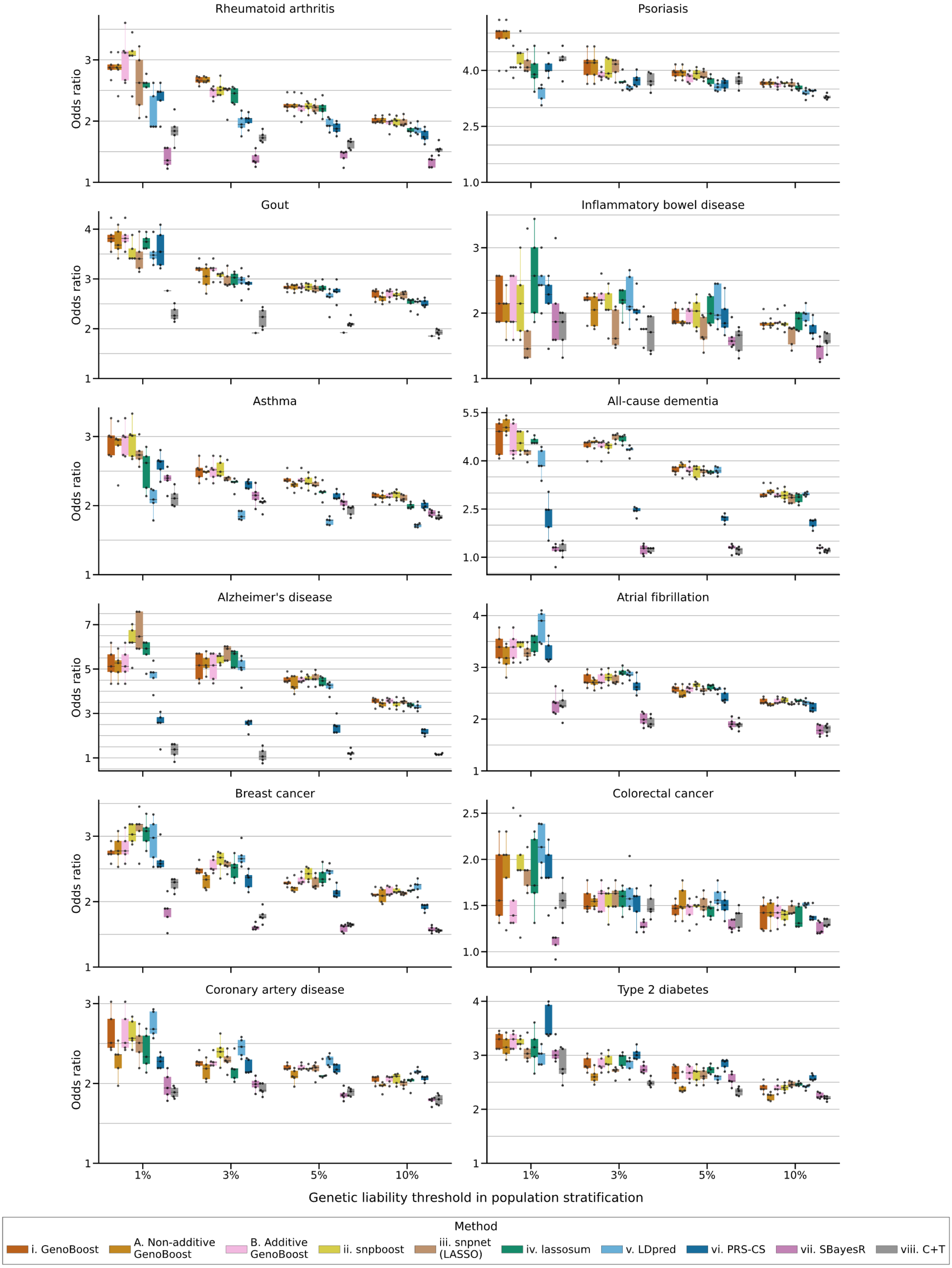

Supplementary Figure 4. **d** Predictive ability (odds ratio) of GenoBoost PGS models in stratifying high-risk within the top 1%-tile (as well as 3, 5, and 10%-tile) vs. the remaining population in the hold-out test set, similar to Fig. 2c. On each box, the center line is the median, the top and bottom of the box are the second and fourth values (Q3 and Q1), and the upper and lower whiskers are shown if the first and the fifth are in Q3 + 1.5 IQR and Q1 – 1.5 IQR, respectively, where IQR = Q3 – Q1. Source data are provided as a Source Data file.

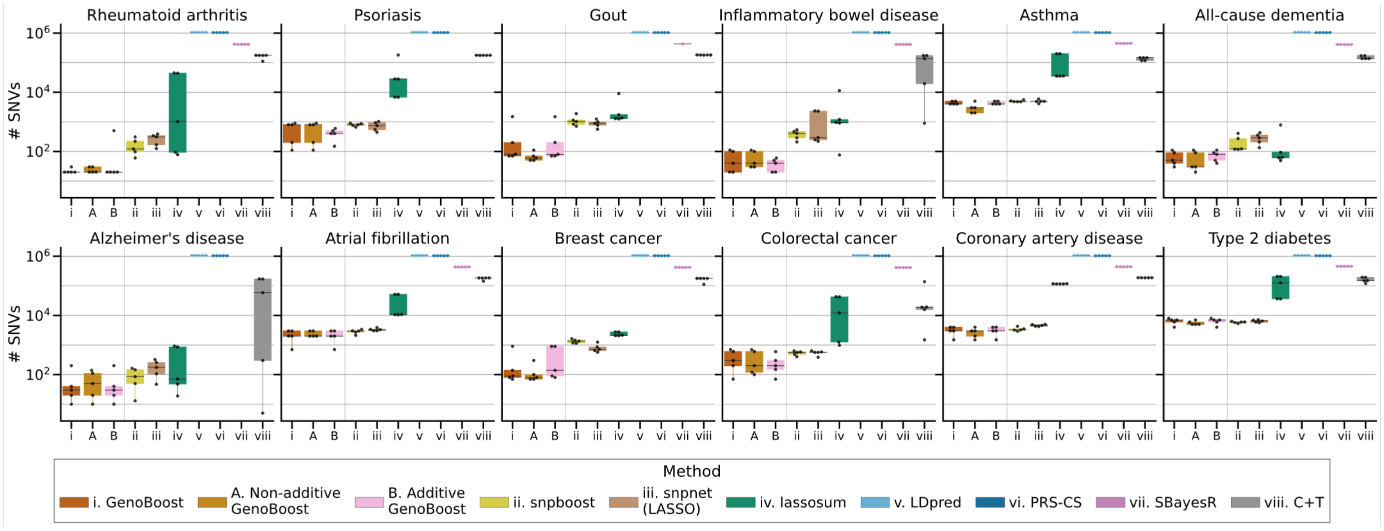

Supplementary Figure 4. **e** Number of selected SNVs in PGS models across GenoBoost against seven commonly used methods across twelve diseases in UK Biobank. The y-axis shows the number of SNVs in a log-scale, similar to Fig. 2d. On each box, the center line is the median, the top and bottom of the box are the second and fourth values (Q3 and Q1), and the upper and lower whiskers are shown if the first and the fifth are in Q3 + 1.5 IQR and Q1 – 1.5 IQR, respectively, where IQR = Q3 – Q1. Source data are provided as a Source Data file.

a

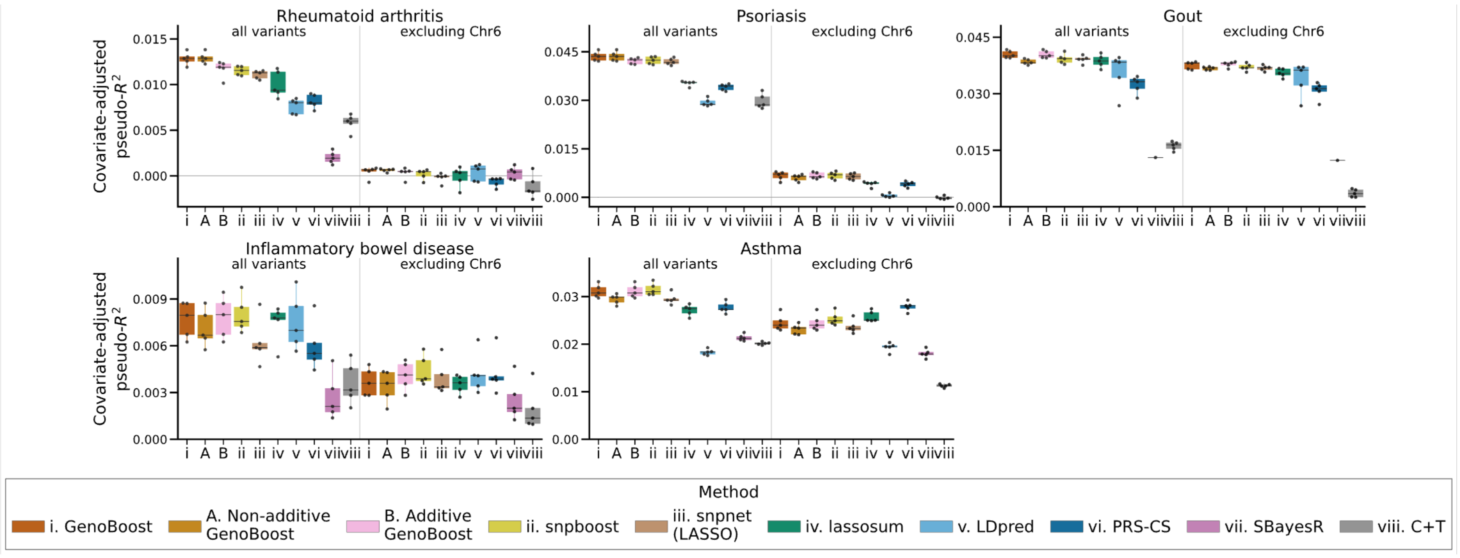

b

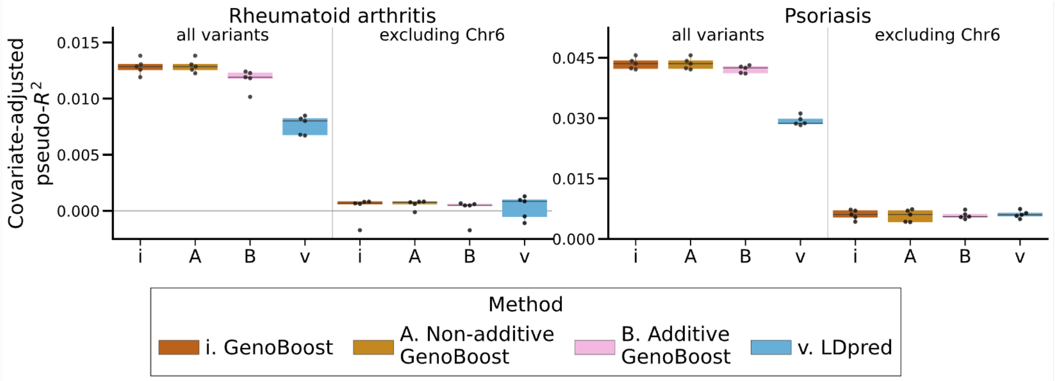

Supplementary Figure 5. **Predictive accuracy of PGS excluding chromosome 6. a** Covariate-adjusted pseudo-R^2 for five diseases in UK Biobank on PGS trained with whole genotype and excluding chromosome 6 in the test dataset. **b** Covariate-adjusted pseudo-R^2 for rheumatoid arthritis and psoriasis on PGS trained with genotype excluding chromosome 6. On each box, the center line is the median, the top and bottom of the box are the second and fourth values (Q3 and Q1), and the upper and lower whiskers are shown if the first and the fifth are in Q3 + 1.5 IQR and Q1 – 1.5 IQR, respectively, where IQR = Q3 – Q1. Source data are provided as a Source Data file.

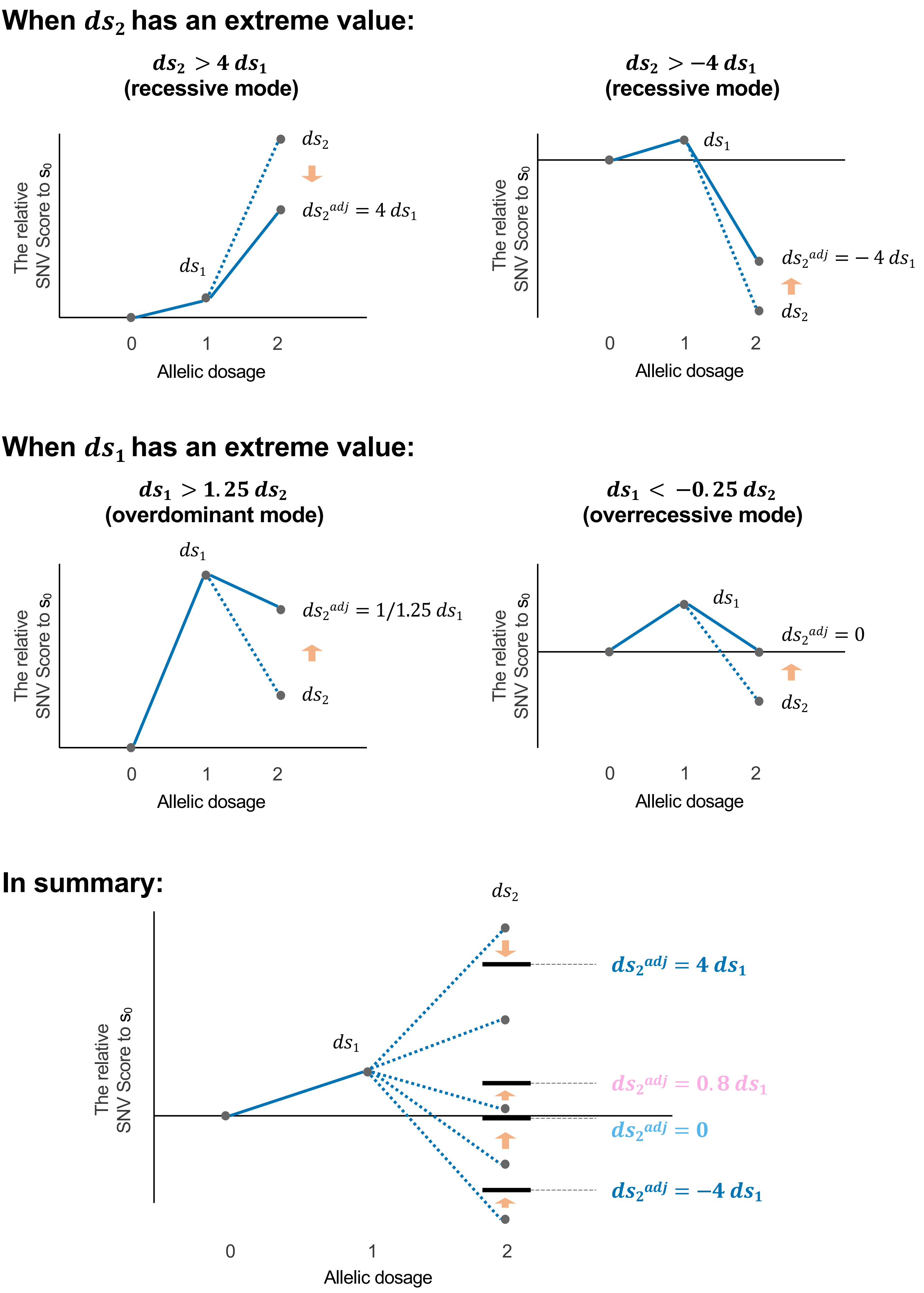

Supplementary Figure 6. **Limiting extremely large absolute SNV scores.** s_2 is adjusted toe the border of genetic modes (Supplementary Fig. 2) when ds_1 (= s_1 - s_0) or ds_2 (= s_2 - s_0) has extremely large value.

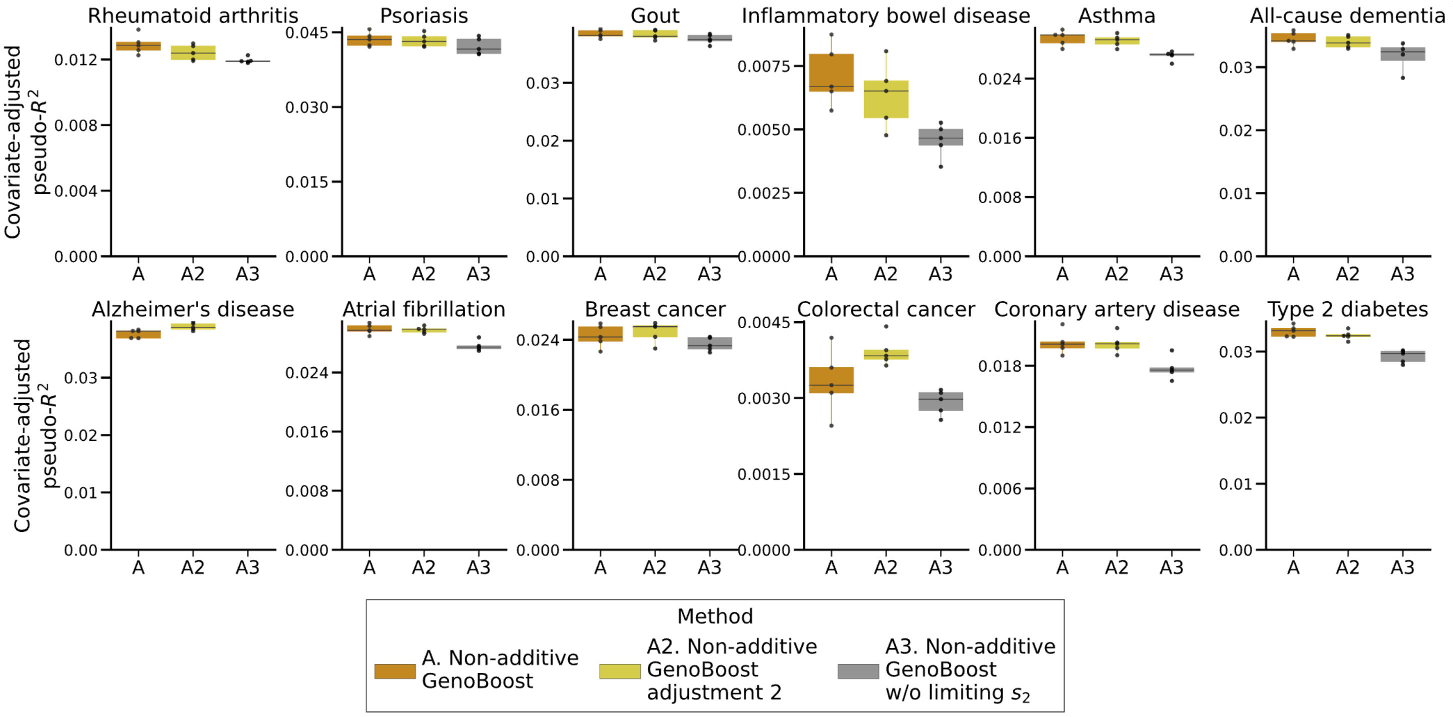

Supplementary Figure 7. **Benchmarking non-additive GenoBoost across twelve diseases in UK Biobank when varying adjustment of s_2.** Covariate-adjusted pseudo-R^2 of non-additive GenoBoost (A) is shown along with one with a different adjustment (A2, Supplementary Methods) and without adjustment (A3). Non-additive GenoBoost without adjustment failed for Alzheimer's disease due to an excessively large s_2. On each box, the center line is the median, the top and bottom of the box are the second and fourth values (Q3 and Q1), and the upper and lower whiskers are shown if the first and the fifth are in Q3 + 1.5 IQR and Q1 – 1.5 IQR, respectively, where IQR = Q3 – Q1. Source data are provided as a Source Data file.

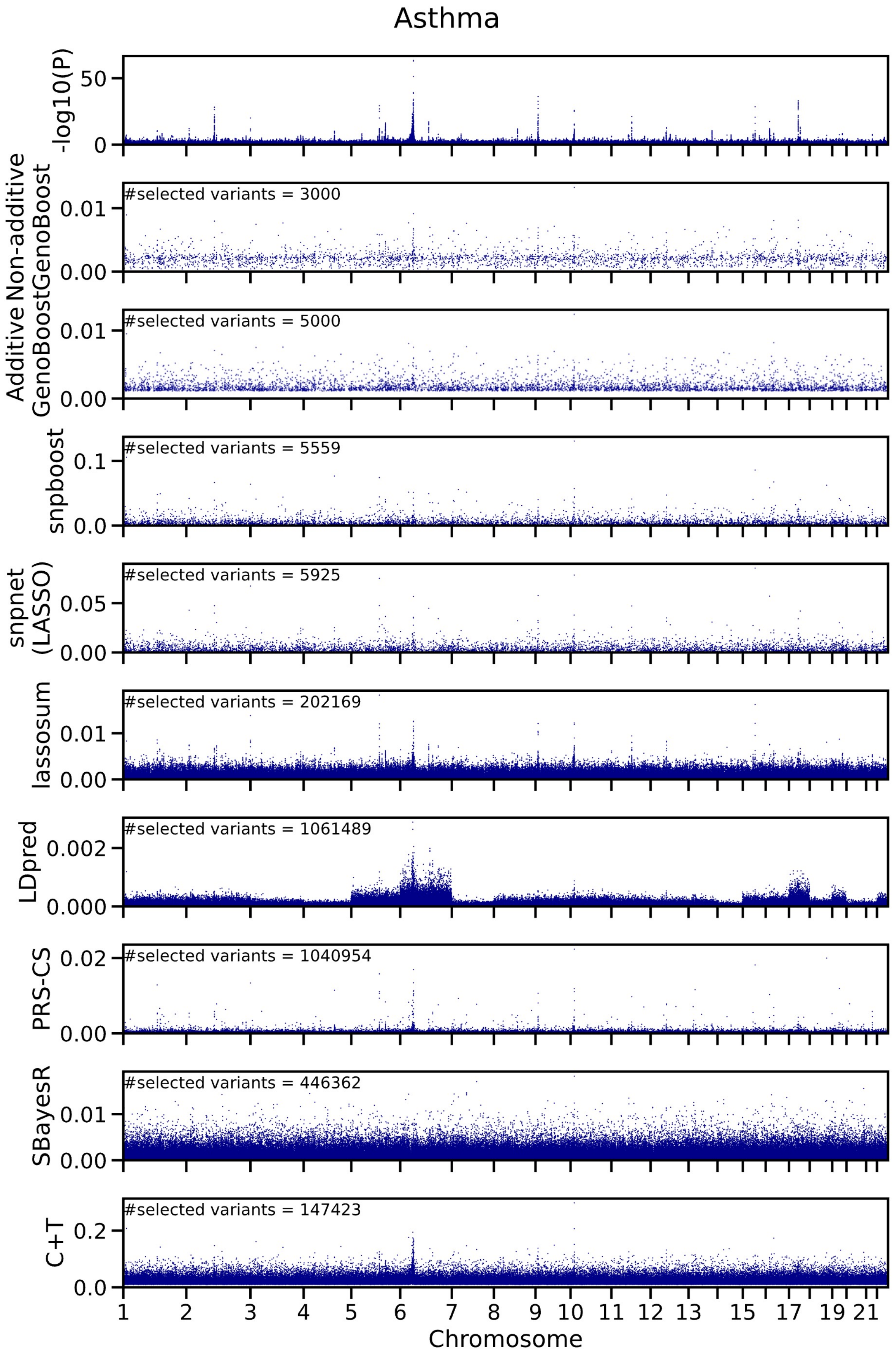

Supplementary Figure 8. **Manhattan plot of GWAS univariate *p*-values and absolute values of multivariate effect sizes estimated from PGS methods. a** For asthma, we calculated *p*-values for GWAS, and absolute values of effect sizes for Non-additive and Additive GenoBoost, snpboost, snpnet, lassosum, LDpred, PRS-CS, SBayesR, and C+T using the best parameter of the primary cross-validation dataset. The *p*-values are by logistic regression (two-sided, no adjustments for multiple comparisons) with *n*=215,768 sample. The effect sizes of Non-additive GenoBoost indicate the relative score of heterozygotes to that of major allele homozygotes ($s_1 - s_0$).

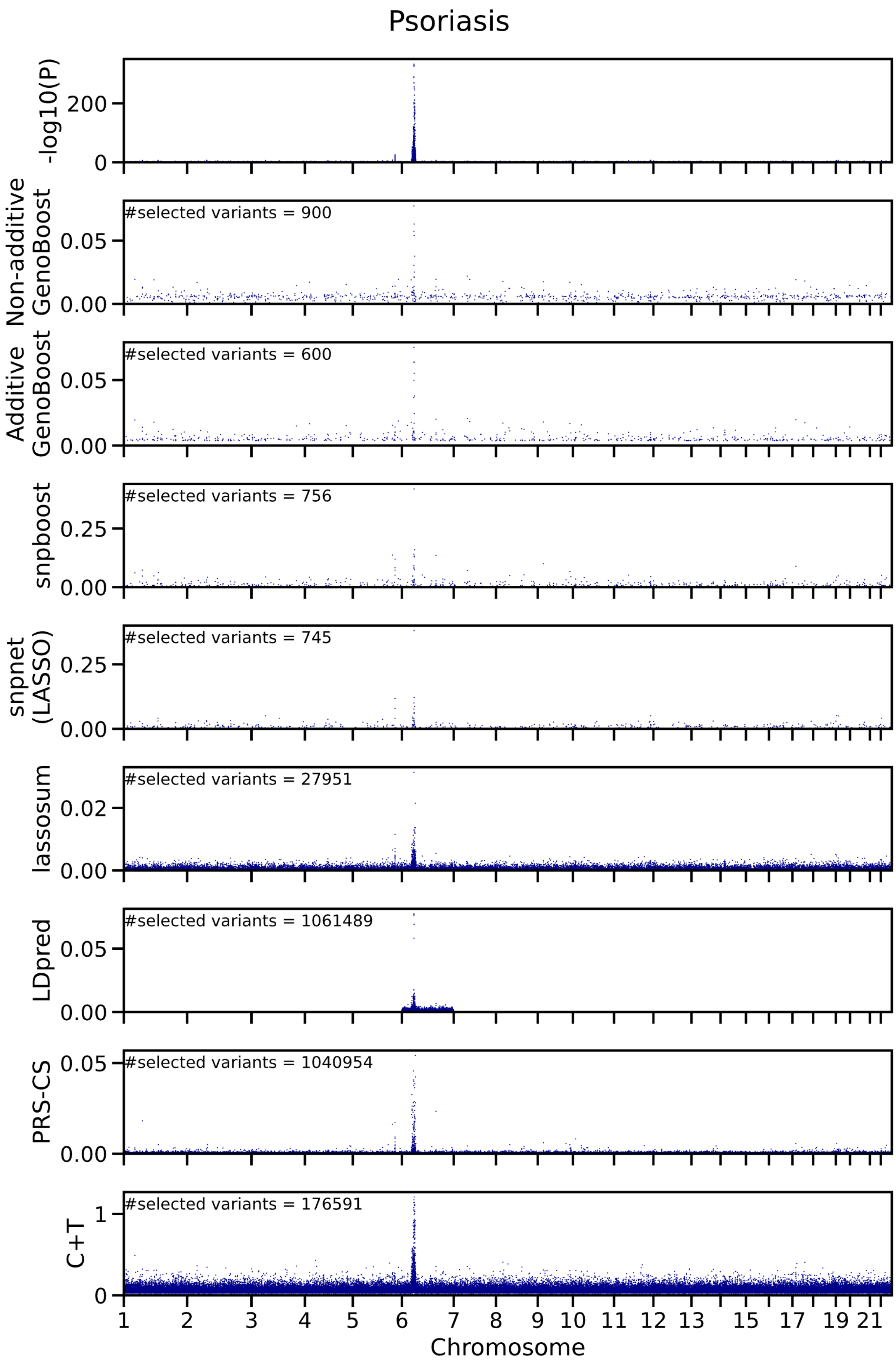

Supplementary Figure 8. **b** The same figure as Supplementary Fig. 8a but for psoriasis.

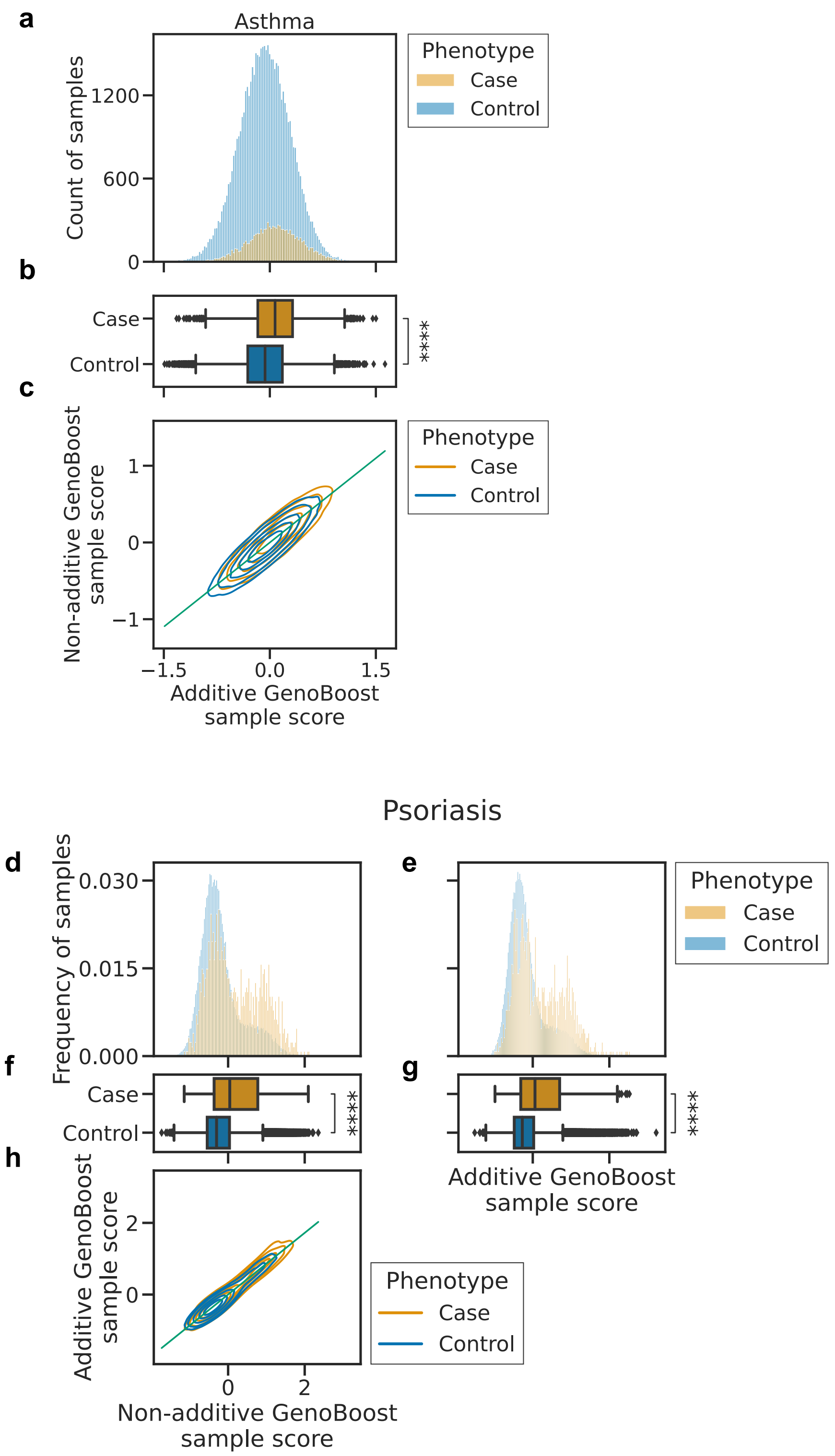

Supplementary Figure 9. **PGS distribution for asthma and psoriasis. a** The Additive GenoBoost polygenic score distributions of asthma case and control samples in the primary hold-out test replicate. **b** Score distributions of case and control samples were statistically different ($p < 1 \times 10^{-200}$; two-sided Mann-Whitney's U test). **c** Comparison of Additive GenoBoost and Non-additive GenoBoost polygenic score distributions. Kernel density plot of the score distributions of Additive GenoBoost on the x-axis and Non-additive GenoBoost on the y-axis for all test samples to show the difference in distributions of case and control. The contours showed kernel density levels of 2%, 5%, 10%, and every 20% from 20% to 100%. The regressed line is also shown. **d-e** The Non-additive GenoBoost (**d**) and Additive GenoBoost (**e**) polygenic score distribution of psoriasis case and control samples in the hold-out test set. **f-g** The Non-additive GenoBoost (**f**) and Additive GenoBoost (**g**) polygenic score distributions of case and control samples were both statistically different ($p < 1 \times 10^{-80}$; two-sided Mann-Whitney's U test). **h** Comparison of Additive GenoBoost and Non-additive GenoBoost polygenic score distributions.

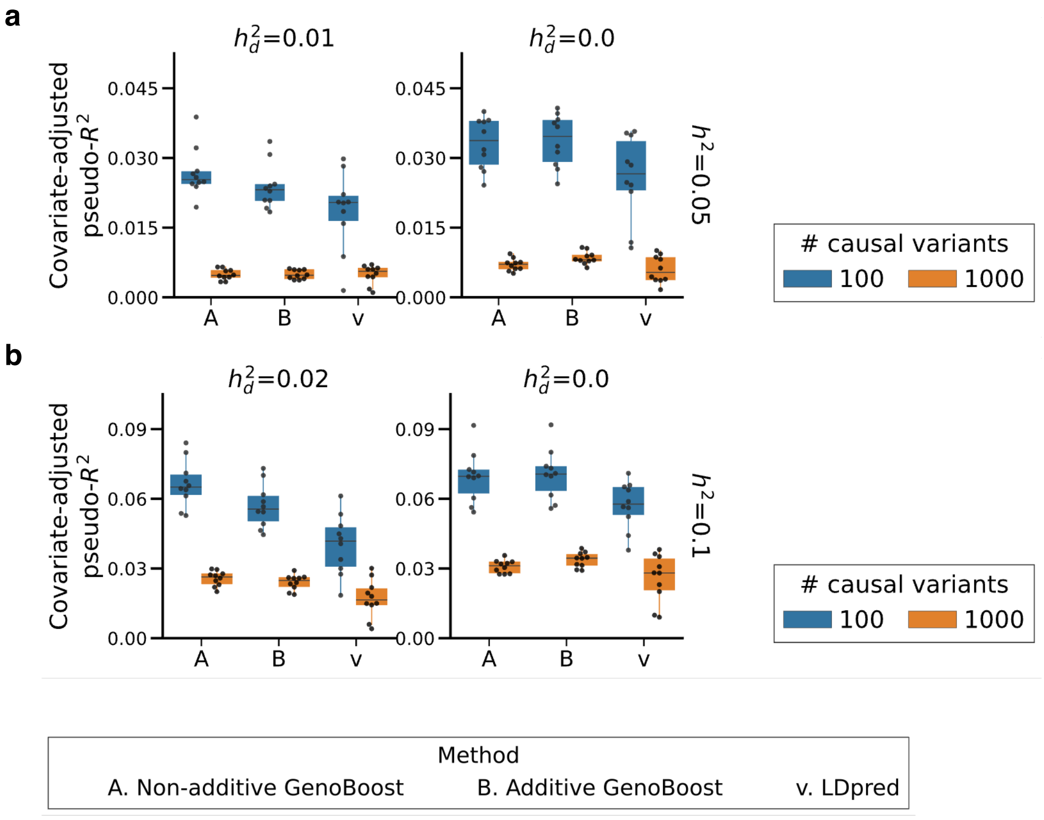

Supplementary Figure 10. **Prediction accuracy on simulation data.** Covariate-adjusted pseudo-R^2 on simulation data with overall heritability (h^2) of 0.05 (**a**), 0.1 (**b**) with dominance heritability (h_d^2) of 20% and 0%, and 100 and 1000 causal SNVs were benchmarked. Ten simulations per parameter are shown along with boxplots (n=10). On each box, the center line is the median, the top and bottom of the box are the first and third quartiles (Q3 and Q1), and the upper and lower whiskers are Q3 + 1.5 IQR and Q1 – 1.5 IQR, respectively, where IQR = Q3 – Q1. Source data are provided as a Source Data file.

a

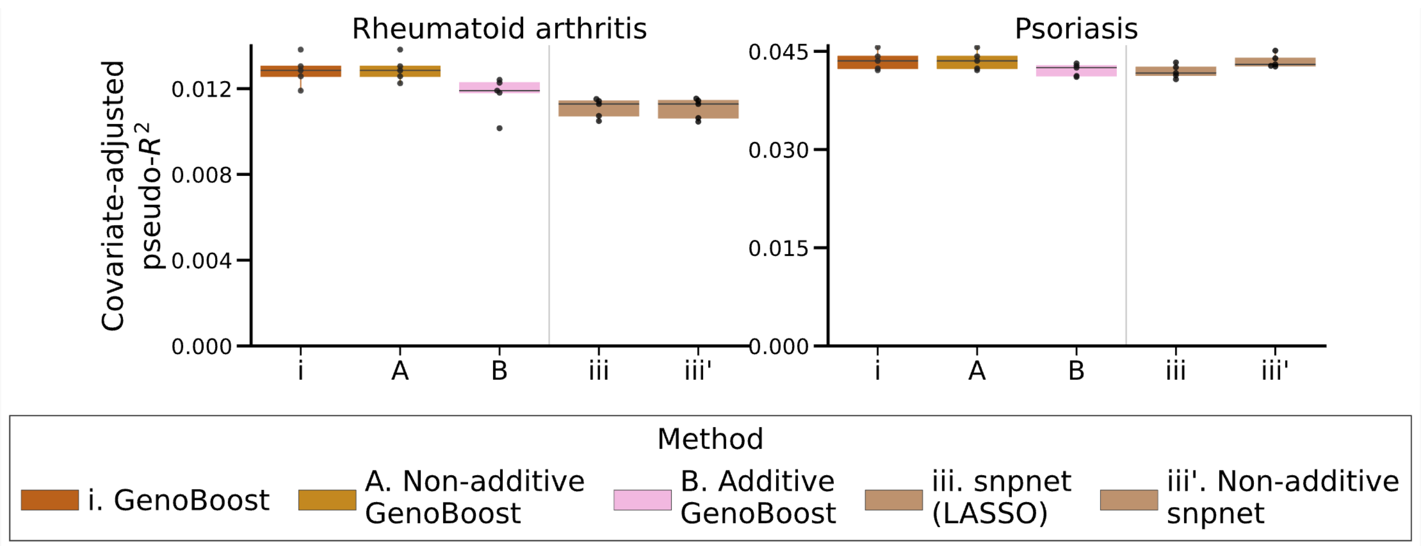

b

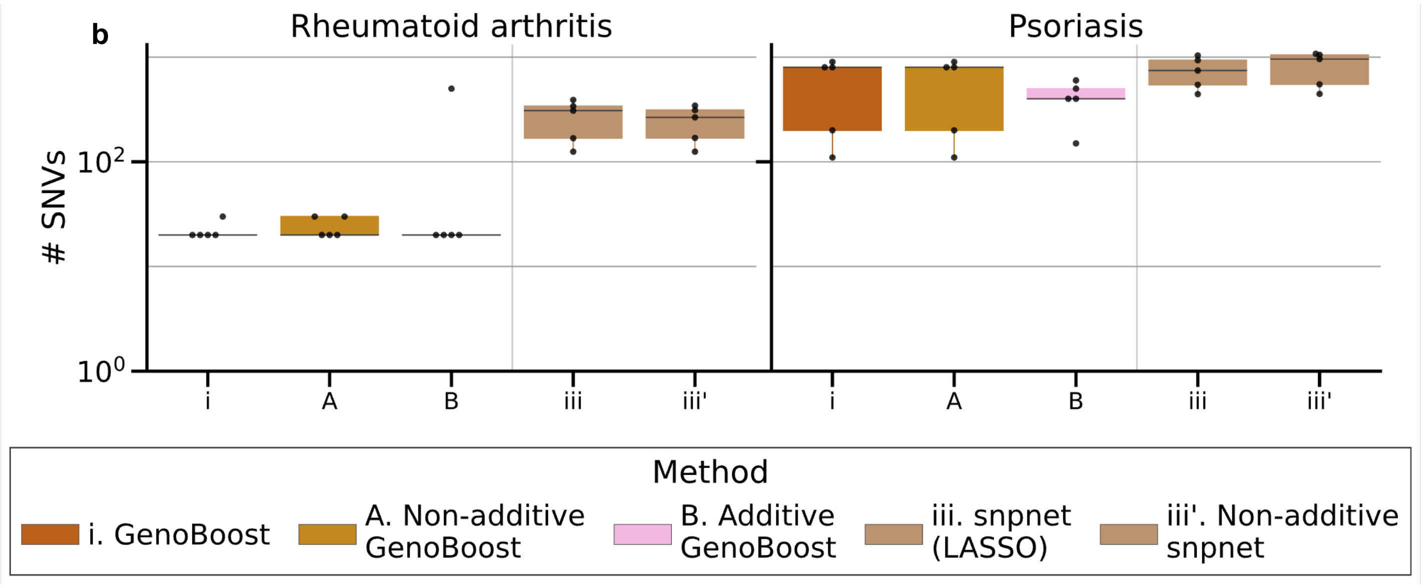

Supplementary Figure 11. **Benchmarking Non-additive snpnet.** Non-additive snpnet [LASSO] was implemented by introducing dummy non-additive genotype in addition to the additive genotype. Covariate-adjusted pseudo-R^2 of PGS (**a**) and the number of SNVs in the PGS (**b**) are shown. On each box, the center line is the median, the top and bottom of the box are the second and fourth values (Q3 and Q1), and the upper and lower whiskers are shown if the first and the fifth are in Q3 + 1.5 IQR and Q1 – 1.5 IQR, respectively, where IQR = Q3 – Q1. Source data are provided as a Source Data file.

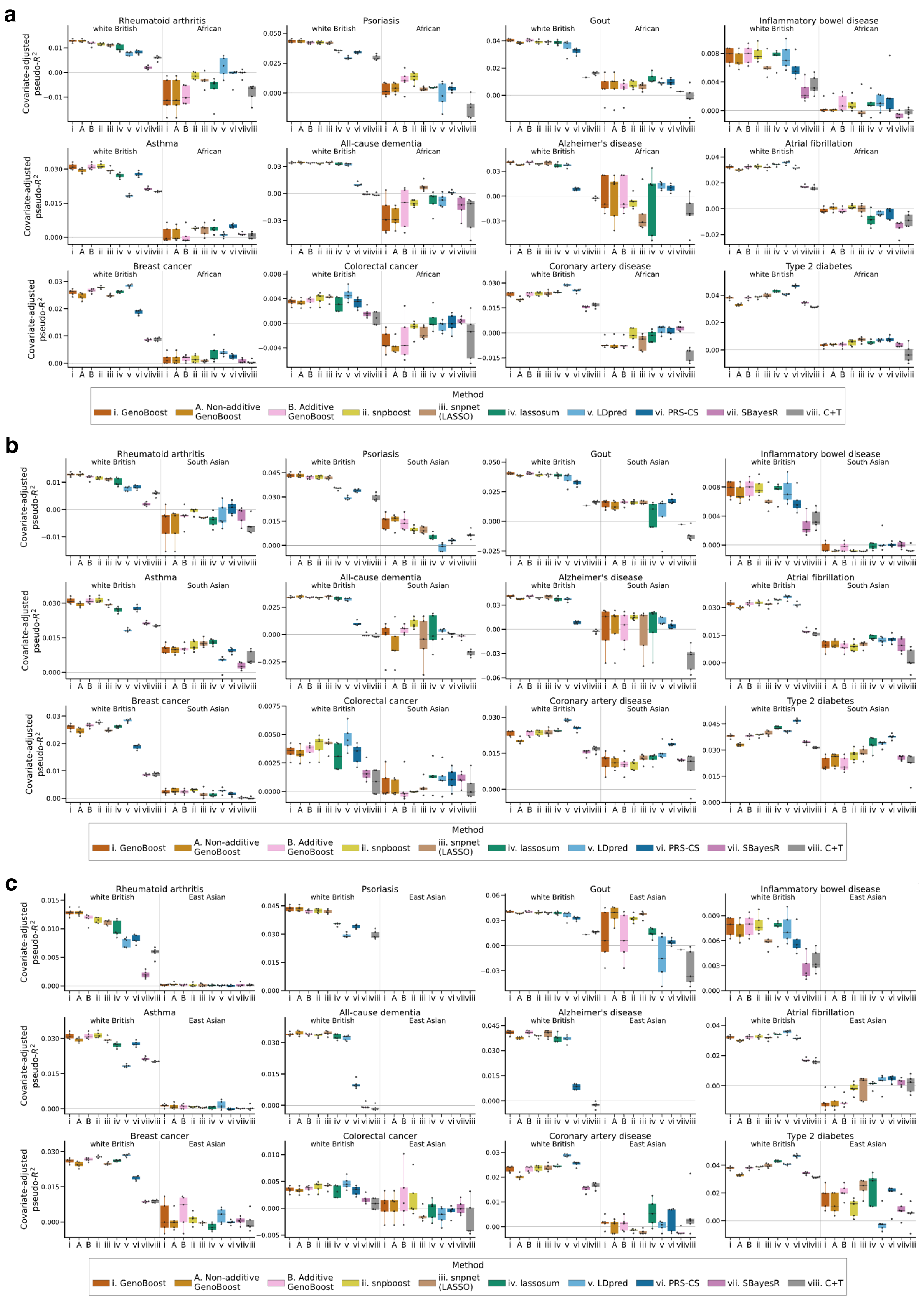

Supplementary Figure 12. **Prediction accuracy for African, South Asian, and East Asian samples compared to white British samples in UK Biobank.** Covariate-adjusted pseudo-R^2 of PGS models across GenoBoost (i, A, B) and seven other methods (ii-viii) for twelve phenotypes for African (**a**, *n*=5,190), South Asian (**b**, *n*=6,362), and East Asian (**c**, *n*=1,416) test samples compared to white British samples were shown. Female samples were used for breast cancer (*n*=2,975; African, *n*=2,867; South Asian, *n*=904; East Asian). Psoriasis, inflammatory bowel disease, all-cause dementia, and Alzheimer's disease for East Asian samples are not shown since regression in validation dataset failed due to small number of case samples. On each box, the center line is the median, the top and bottom of the box are the second and fourth value (Q3 and Q1), the upper and lower whiskers are shown if the first and the fifth are in Q3 + 1.5 IQR and Q1 – 1.5 IQR, respectively, where IQR = Q3 – Q1. Source data are provided as a Source Data file.

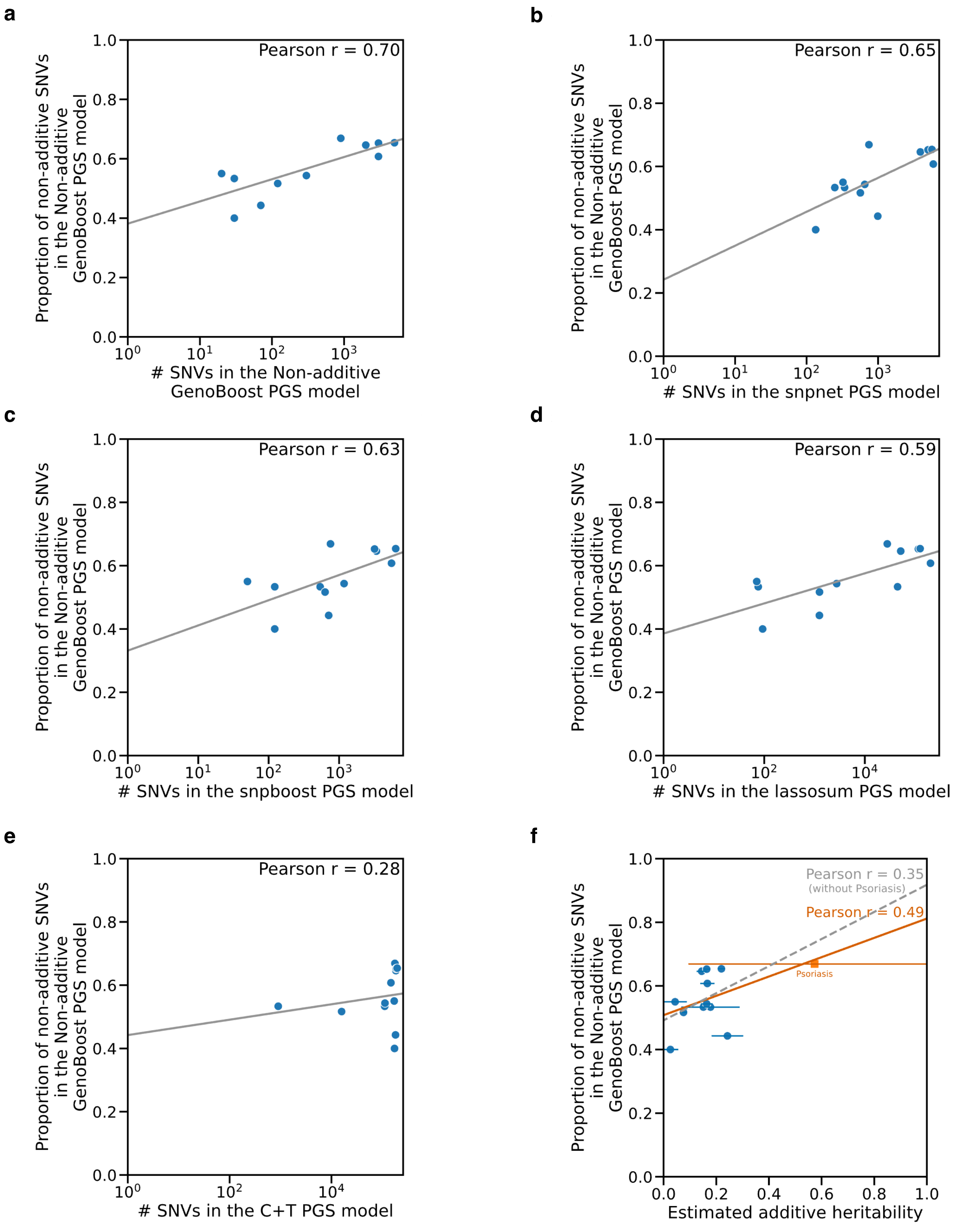

Supplementary Figure 13. **Relationship between the polygenicity or the estimated heritability and the proportion of non-additive SNV effects among the SNVs in the Non-additive GenoBoost PGS model.** The number of SNVs in the PGS models (x-axis) from Non-additive GenoBoost (**a**), snpnet (**b**), snpboost (**c**), lassosum (**d**), and C+T (**e**) and the proportion of SNVs with non-additive genetic dominance effects in the Non-additive GenoBoost PGS model (y-axis) are shown. The estimated additive heritability with standard error (x-axis) and the proportion of SNV with estimated non-additive genetic dominance effects (y-axis) is also shown (**f**). A gray line represents linear regression fit. We show Pearson's correlations in each comparison in the plot. For panel (**f**), we analyzed all phenotypes (gray) as well as eleven phenotypes psoriasis (orange). Source data are provided as a Source Data file.

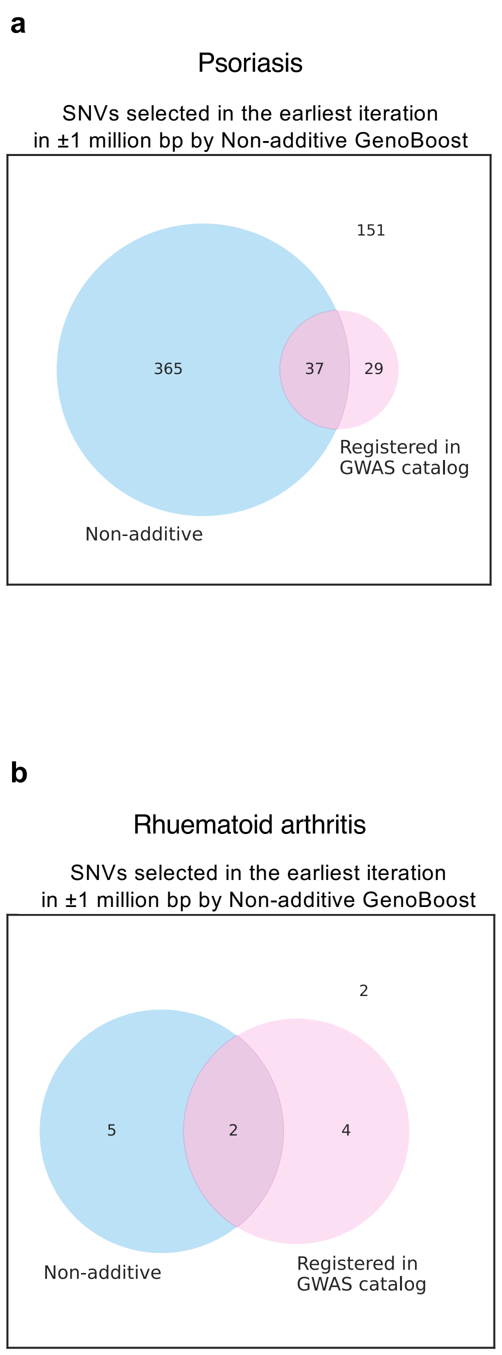

Supplementary Figure 14. **Overlap of SNVs with non-additive effects and reported SNVs in GWAS catalog.** The SNVs selected in the earliest iteration in ±1 million bp by Non-additive GenoBoost were classified in two perspectives: genetic inheritance mode and whether SNVs within ±1 million bp were reported in GWAS catalog. SNVs in Figure 4 for psoriasis were the first five SNVs in or close to genes out of SNVs with non-additive effects and not reported SNVs in GWAS catalog.

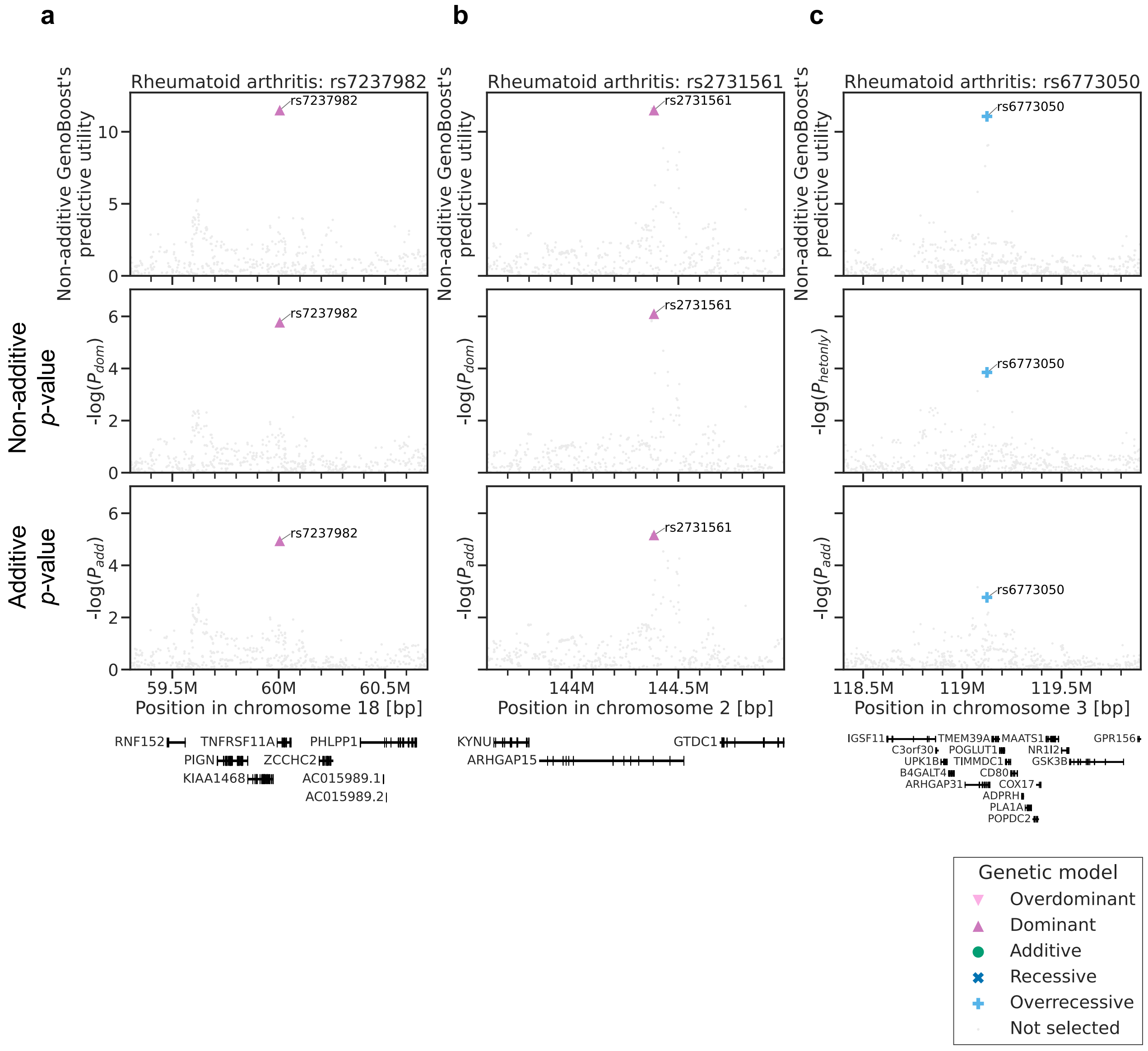

Supplementary Figure 15. **Locus zoom plot of neighboring regions of non-additive SNV effects in Figure 4 for rheumatoid arthritis.** Non-additive GenoBoost's predictive utility, additive and non-additive *p*-values of selected SNVs (colored) and neighboring ones (gray) are shown for rheumatoid arthritis. Colored and annotated variants are the first selected variants in the neighboring 1 million bp shown in Figure 4. The regions are ordered by non-additive GenoBoost selection order. The *p*-values are by logistic regression (two-sided, no adjustments for multiple comparisons) with *n*=215,768 sample.

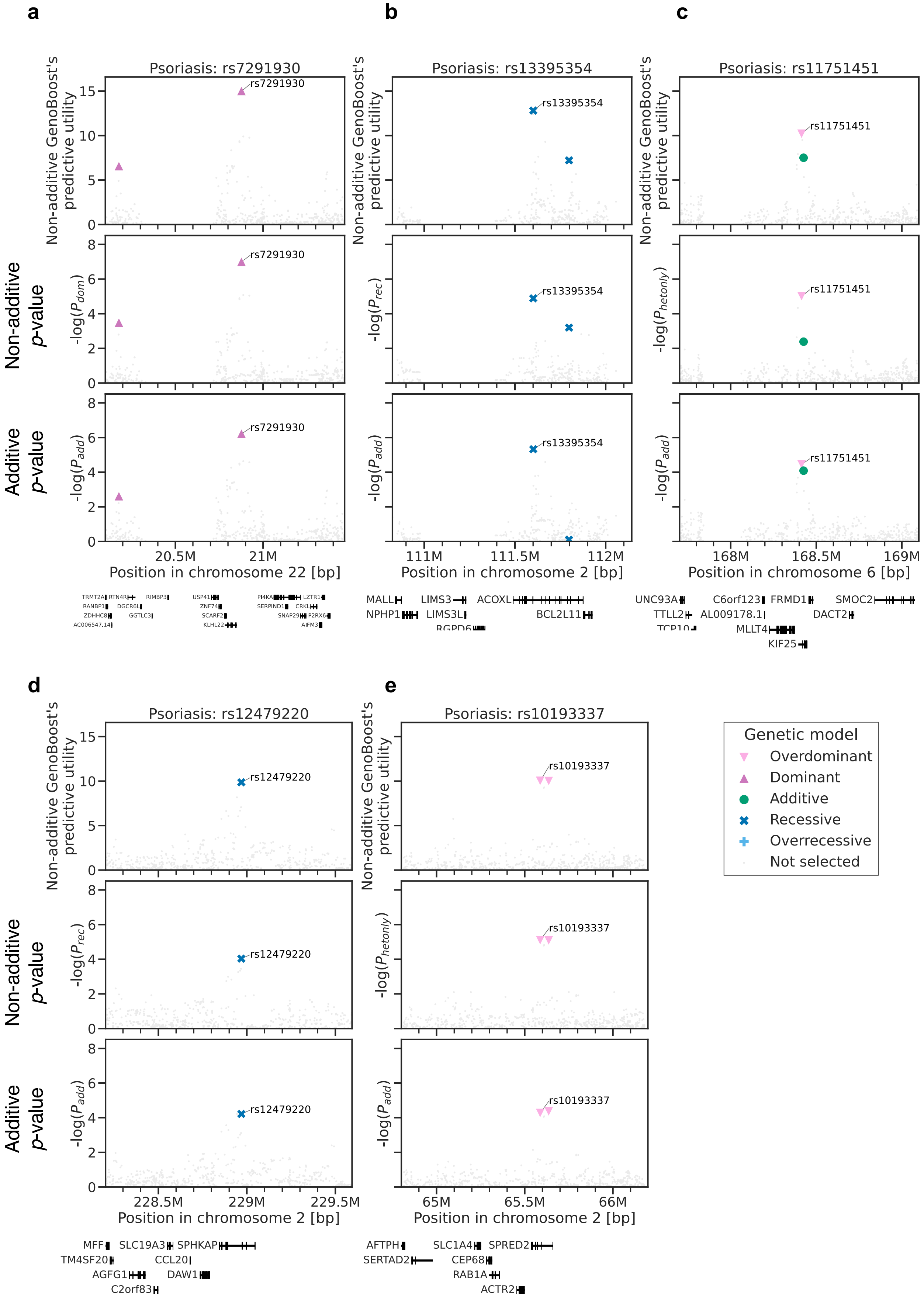

Supplementary Figure 16. **Locus zoom plot of neighboring regions of non-additive SNV effects in Figure 4 for psoriasis.** The same figure as Supplementary Fig. 15 but for psoriasis.

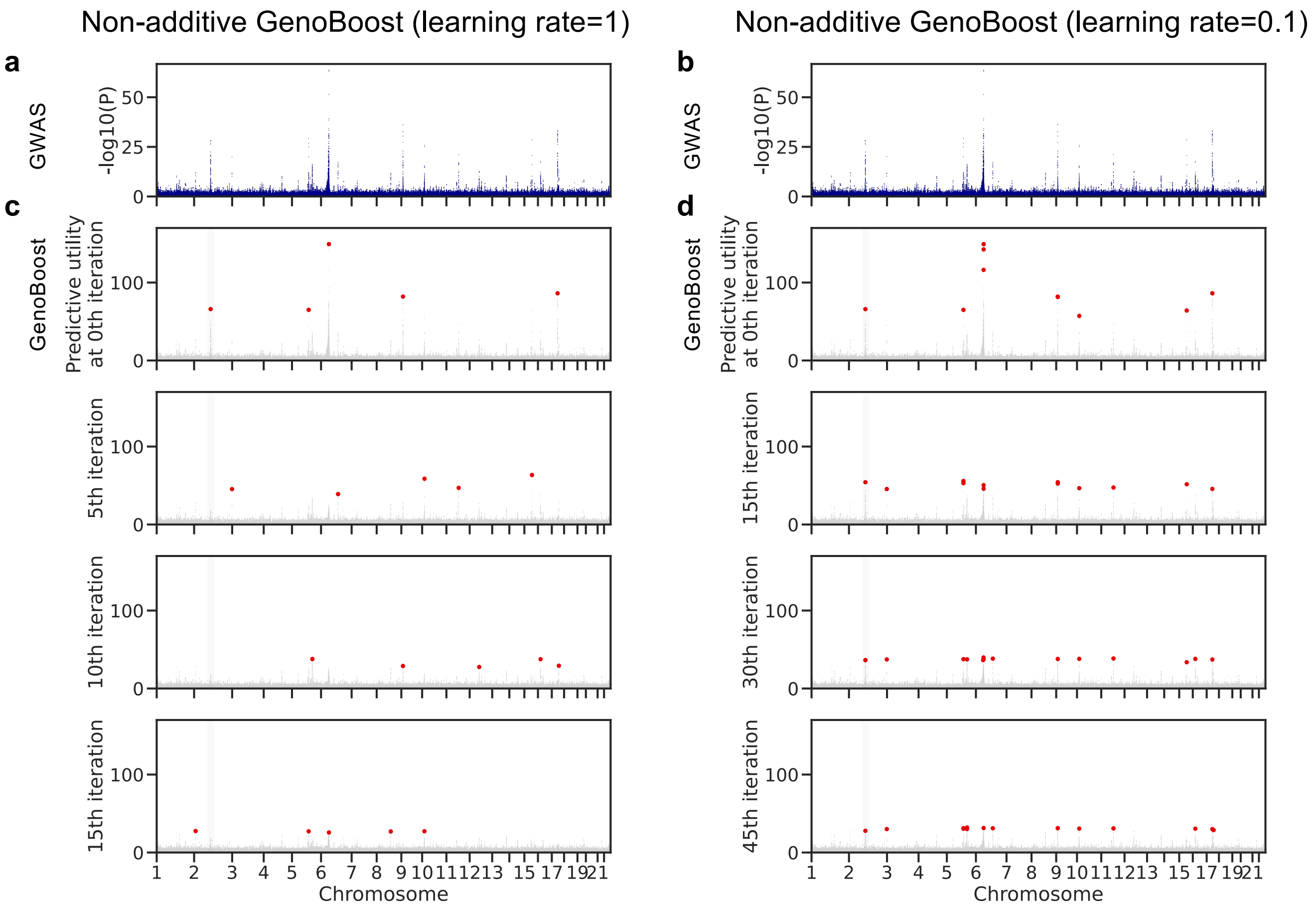

Supplementary Figure 17. **Manhattan plot and plot of predictive utility of asthma for non-additive GenoBoost without and with a learning rate. a-b** Manhattan plots show additive *p*-values of asthma by logistic regression (two-sided, no adjustments for multiple comparisons) with *n*=215,768 samples on the primary training dataset. **c-d** The predictive utility, where an SNV with a larger predictive utility means the more associated SNV to the phenotype for GenoBoost without a learning rate by setting learning rate 1 (**c**) and GenoBoost with a learning rate of 0.1 (**d**). Red dots highlight SNVs selected in the next five iterations for GenoBoost without a learning rate and the next 15 iterations for GenoBoost with a learning rate. Underlying gray dots show unselected SNVs. SNV predictive utility at the first iteration was generally similar to the *p*-value. GenoBoost without a learning rate suggests that only one SNV could explain the variance in the region with the gray background, but GenoBoost with a learning rate selects several times from the region.

a

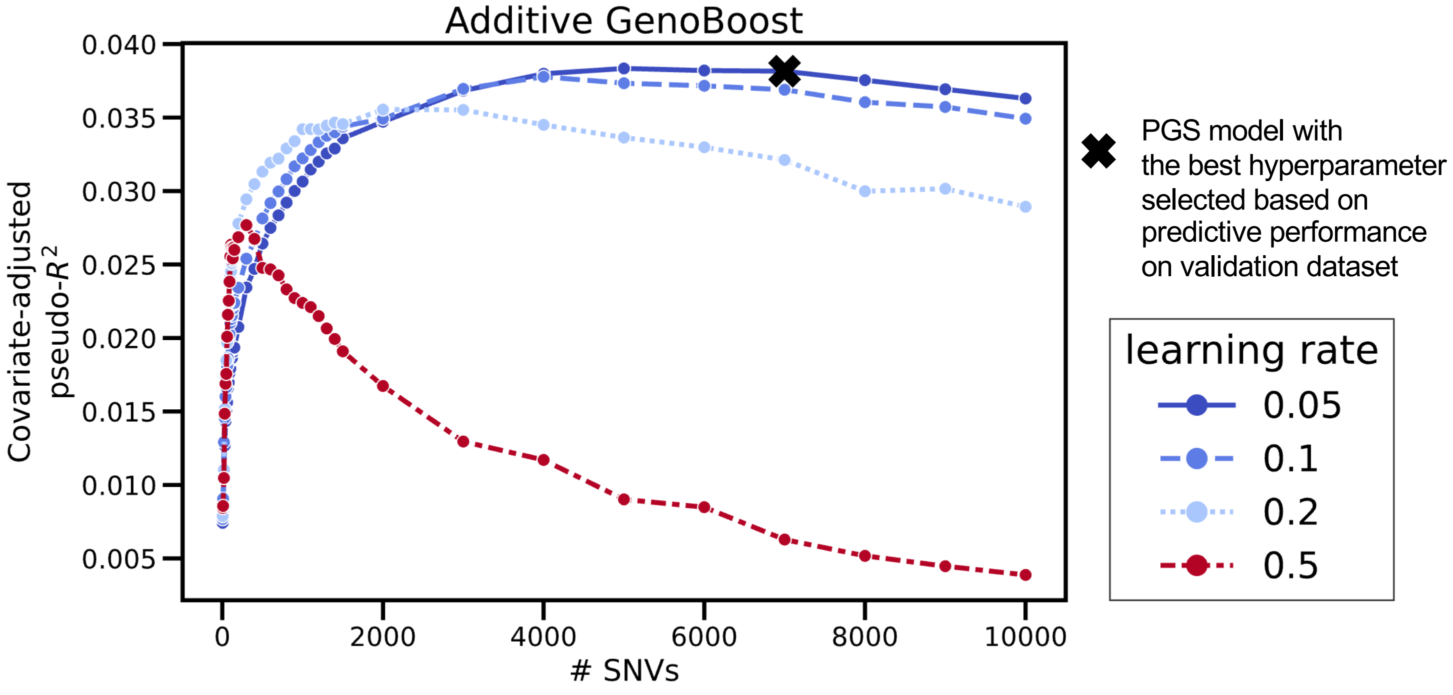

b

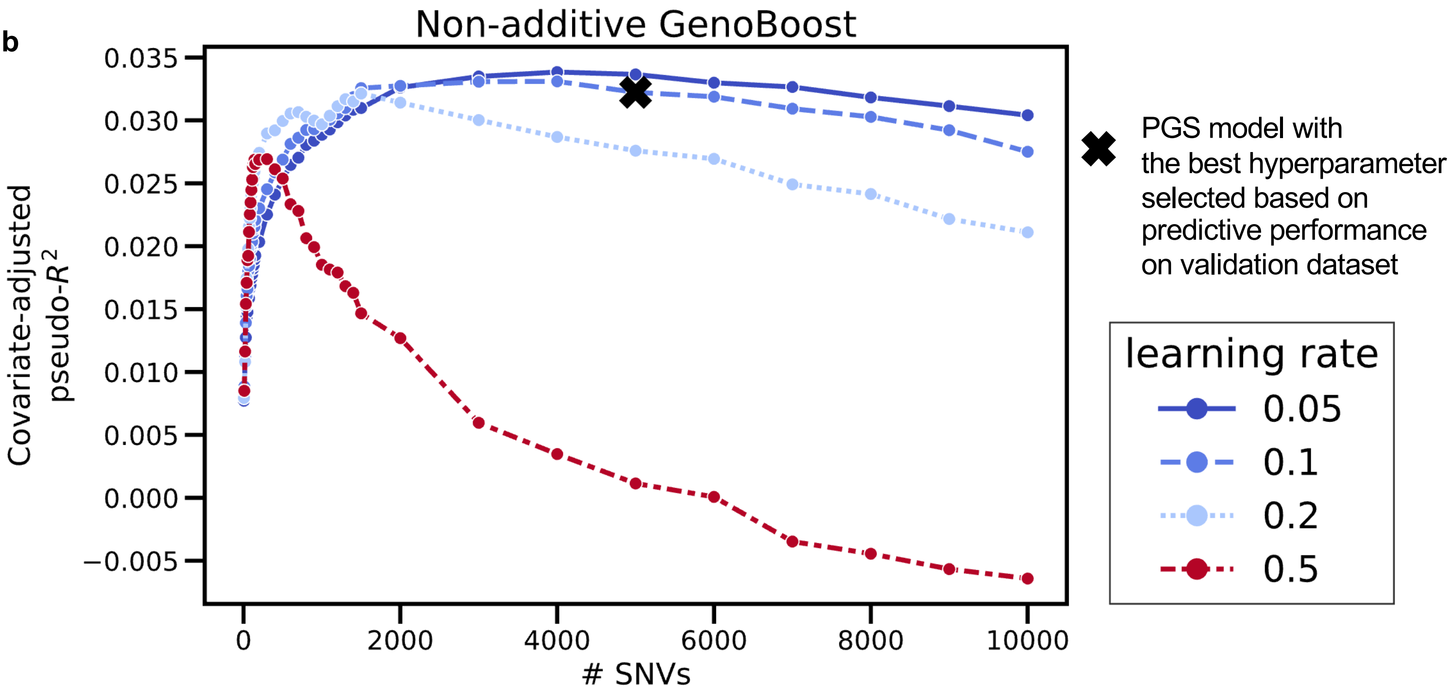

Supplementary Figure 18. **Prediction Accuracy for parameter candidates of GenoBoost.** Covariate-adjusted pseudo-R^2 of the test dataset for each parameter candidate and the best parameter of additive GenoBoost (a) and non-additive GenoBoost (b) for Type 2 Diabetes. The best parameter was determined in the validation dataset.

Phenotype	Variant	MAF [%]	Gene			GenoBoost	Association Test	
			Nearest gene	Variant type	Reference (PubMed ID) to suggest the association of gene to phenotype	inheritance mode inferred by GenoBoost	*P*-value under genetic model inferred by GenoBoost	Additive *p*-value
Rheumatoid arthritis	rs7237982	24	*TNFRSF11A (RANK)*	Intron	32149122, 17341304	Dominant	1.7e-6	1.1e-5
	rs2731561	25	*ARHGAP15*	Intron	26359667	Dominant	8.2e-7	7.0e-6
	rs6773050	47	*ARHGAP31*	Intron	-	Overrecessive	1.5e-4	1.7e-3
Psoriasis	rs7291930	23	*MED15*	Intron	30061880	Dominant	1.0e-7	6.2e-7
	rs13395354	18	*ACOXL*	Intron	-	Recessive	1.3e-5	4.8e-6
	rs11751451	14	*KIF25*	Intron	-	Overdominant	9.8e-6	3.7e-5
	rs12479220	23	*SPHKAP*	Intron	-	Recessive	9.3e-5	6.2e-5
	rs10193337	5.2	*SPRED2*	Intron	26086874	Overdominant	7.9e-6	4.7e-4

Supplementary Table 7. **Association *p*-values of non-additive SNV effects in or close to genes included in the GenoBoost PGS model.** In addition to Figure 4, *p*-values by logistic regression (two-sided, no adjustments for multiple comparisons) with *n*=215,768 samples under additive model and genetic model inferred by GenoBoost are shown. *MAF,* minor allele frequency.

Supplementary References

[1] Friedman, J., Hastie, T. & Tibshirani, R. Additive logistic regression: a statistical view of boosting (with discussion and a rejoinder by the authors). *aos* **28**, 337–407 (2000).

[2] Schapire, R. E. & Freund, Y. *Boosting: Foundations and Algorithms* (The MIT Press, Cambridge, MA, 2012).

[3] Friedman, J. H. Greedy function approximation: A gradient boosting machine. *aos* **29**, 1189–1232 (2001).

[4] Qian, J. *et al.* A fast and scalable framework for large-scale and ultrahigh-dimensional sparse regression with application to the UK biobank. *PLoS Genet.* **16**, e1009141 (2020).

[5] Klinkhammer, H., Staerk, C., Maj, C., Krawitz, P. M. & Mayr, A. A statistical boosting framework for polygenic risk scores based on large-scale genotype data. *Front. Genet.* **13**, 1076440 (2022).

[6] Chang, C. C. *et al.* Second-generation PLINK: rising to the challenge of larger and richer datasets. *Gigascience* **4**, 7 (2015).

[7] Bycroft, C. *et al.* The UK biobank resource with deep phenotyping and genomic data. *Nature* **562**, 203–209 (2018).

[8] Topless, R. K. *et al.* Gout, rheumatoid arthritis, and the risk of death related to coronavirus disease 2019: An analysis of the UK biobank. *ACR Open Rheumatol* **3**, 333–340 (2021).

[9] Patrick, M. T. *et al.* Shared genetic risk factors and causal association between psoriasis and coronary artery disease. *Nat. Commun.* **13**, 6565 (2022).

[10] Khera, A. V. *et al.* Genome-wide polygenic scores for common diseases identify individuals with risk equivalent to monogenic mutations. *Nat. Genet.* **50**, 1219–1224 (2018).

[11] Gong, J., Harris, K., Peters, S. A. E. & Woodward, M. Sex differences in the association between major cardiovascular risk factors in midlife and dementia: a cohort study using data from the UK biobank. *BMC Med.* **19**, 110 (2021).

[12] Arthur, R. S., Dannenberg, A. J., Kim, M. & Rohan, T. E. The association of body fat composition with risk of breast, endometrial, ovarian and colorectal cancers among normal weight participants in the UK biobank. *Br. J. Cancer* **124**, 1592–1605 (2021).

[13] Sinnott-Armstrong, N. *et al.* Genetics of 35 blood and urine biomarkers in the UK biobank. *Nat. Genet.* **53**, 185–194 (2021).

[14] Mak, T. S. H., Porsch, R. M., Choi, S. W., Zhou, X. & Sham, P. C. Polygenic scores via penalized regression on summary statistics. *Genet. Epidemiol.* **41**, 469–480 (2017).

[15] Vilhjálmsson, B. J. *et al.* Modeling linkage disequilibrium increases accuracy of polygenic risk scores. *Am. J. Hum. Genet.* **97**, 576–592 (2015).

[16] Ge, T., Chen, C.-Y., Ni, Y., Feng, Y.-C. A. & Smoller, J. W. Polygenic prediction via bayesian regression and continuous shrinkage priors. *Nat. Commun.* **10**, 1776 (2019).

[17] Lloyd-Jones, L. R. *et al.* Improved polygenic prediction by bayesian multiple regression on summary statistics. *Nat. Commun.* **10**, 5086 (2019).

[18] International Schizophrenia Consortium *et al.* Common polygenic variation contributes to risk of schizophrenia and bipolar disorder. *Nature* **460**, 748–752 (2009).
