# Supplementary material for: A polygenic score method boosted by non-additive models
Source: Nat Commun. 2024 May 29;15:4433. doi: 10.1038/s41467-024-48654-x (PMC11522481; doi:10.1038/s41467-024-48654-x)
Supplement: Supplementary file 3 — Description of Additional Supplementary Files [file 41467_2024_48654_MOESM3_ESM.pdf]

Description of Additional Supplementary Files

File name: Supplementary Data 1

Description: Statistical Source Data for Supplementary Figures 4, 5, 7, 10-13, 18.

File name: Supplementary Data 2

Description: The proportion of non-additive SNVs in Non-additive GenoBoost PGS model, the number of SNVs in the PGS models from Non-additive GenoBoost, snpnet, snpboost, lassosum, C+T, LDpred, PRS-CS, and SBayesR, and the estimated additive heritability in liability scale on the primary fold for twelve UK Biobank phenotypes.

File name: Supplementary Data 3

Description: The list of all non-additive SNVs in Non-additive GenoBoost PGS model for psoriasis but not reported in GWAS catalog. The SNVs selected first in 1 million bp were shown. The top 5 SNVs were shown for psoriasis in Figure 4.
