# Supplementary material for: A polygenic score method boosted by non-additive models
Source: Nat Commun. 2024 May 29;15:4433. doi: 10.1038/s41467-024-48654-x (PMC11522481; doi:10.1038/s41467-024-48654-x)
Supplement: Supplementary file 7 — Reporting Summary [file 41467_2024_48654_MOESM7_ESM.pdf]

nature portfolio

Corresponding author(s): Rikifumi Ohta, Yosuke Tanigawa, Manolis Kellis, Shinichi Morishita

Last updated by author(s): 4/26/2024

Reporting Summary

Nature Portfolio wishes to improve the reproducibility of the work that we publish. This form provides structure for consistency and transparency in reporting. For further information on Nature Portfolio policies, see our Editorial Policies and the Editorial Policy Checklist.

Statistics

For all statistical analyses, confirm that the following items are present in the figure legend, table legend, main text, or Methods section.

n/a	Confirmed	
☐	☒	The exact sample size (*n*) for each experimental group/condition, given as a discrete number and unit of measurement
☐	☒	A statement on whether measurements were taken from distinct samples or whether the same sample was measured repeatedly
☐	☒	The statistical test(s) used AND whether they are one- or two-sided<br>*Only common tests should be described solely by name; describe more complex techniques in the Methods section.*
☐	☒	A description of all covariates tested
☐	☒	A description of any assumptions or corrections, such as tests of normality and adjustment for multiple comparisons
☐	☒	A full description of the statistical parameters including central tendency (e.g. means) or other basic estimates (e.g. regression coefficient) AND variation (e.g. standard deviation) or associated estimates of uncertainty (e.g. confidence intervals)
☐	☒	For null hypothesis testing, the test statistic (e.g. *F*, *t*, *r*) with confidence intervals, effect sizes, degrees of freedom and *P* value noted<br>*Give P values as exact values whenever suitable.*
☒	☐	For Bayesian analysis, information on the choice of priors and Markov chain Monte Carlo settings
☒	☐	For hierarchical and complex designs, identification of the appropriate level for tests and full reporting of outcomes
☐	☒	Estimates of effect sizes (e.g. Cohen's *d*, Pearson's *r*), indicating how they were calculated

Our web collection on statistics for biologists contains articles on many of the points above.

Software and code

Policy information about availability of computer code

Data collection	No software was used.
Data analysis	GenoBoost (v1.0.8): https://github.com/rickyota/genoboost<br>GenoBoost script (v1.0.0): https://github.com/rickyota/genoboost-paper-script<br>Plink1.9: https://www.cog-genomics.org/plink/<br>Plink2.0: https://www.cog-genomics.org/plink/2.0/<br>snpboost (v0): https://github.com/hklinkhammer/snpboost<br>snpnet (v20221122): https://github.com/junyangq/snpnet<br>lassosum (v0.4.5): https://github.com/tshmak/lassosum<br>LDpred (v1.0.11): https://github.com/bvilhjal/ldpred<br>PRS-CS (v20210604): https://github.com/getian107/PRScs<br>SBayesR (v2.02): https://cnsgenomics.com/software/gctb/#Download<br>Ensembl Variant Effect Predictor (v110): https://www.ensembl.org/vep<br>d-ldsc (v0.1): https://github.com/astheeggeggs/d-ldsc

For manuscripts utilizing custom algorithms or software that are central to the research but not yet described in published literature, software must be made available to editors and reviewers. We strongly encourage code deposition in a community repository (e.g. GitHub). See the Nature Portfolio guidelines for submitting code & software for further information.

Data

Policy information about availability of data

All manuscripts must include a data availability statement. This statement should provide the following information, where applicable:

- Accession codes, unique identifiers, or web links for publicly available datasets
- A description of any restrictions on data availability
- For clinical datasets or third party data, please ensure that the statement adheres to our policy

The analyses presented in this study were based on the individual-level data accessed through UK Biobank: https://www.ukbiobank.ac.uk. This research was conducted using the UK Biobank Resource under Application Number 48405. We used the reference panels from the 1000 genomes project (https://www.internationalgenome.org/) and the list of genetic variants from the HapMap3 projects (https://www.broadinstitute.org/medical-and-population-genetics/hapmap-3). The PGS model weights generated from this study are publicly available in the PGS catalog (publication ID: PGP000546). The experimental data generated in this study have been deposited in the Zenodo database under accession code (https://doi.org/10.5281/zenodo.10200754). Source data are provided with this paper.

Research involving human participants, their data, or biological material

Policy information about studies with human participants or human data. See also policy information about sex, gender (identity/presentation), and sexual orientation and race, ethnicity and racism.

Reporting on sex and gender	Sex was used as covariates on calculating the polygenic score function and genome-wide association study.
Reporting on race, ethnicity, or other socially relevant groupings	White British individuals and non-European ancestry smaples were used for the analyses.
Population characteristics	Sex, age and principal components (PC1-10) were used as covariates on calculating the polygenic score function and genome-wide association study.
Recruitment	N/A (Performed by UK Biobank)
Ethics oversight	N/A (Performed by UK Biobank)

Note that full information on the approval of the study protocol must also be provided in the manuscript.

Field-specific reporting

Please select the one below that is the best fit for your research. If you are not sure, read the appropriate sections before making your selection.

☒ Life sciences ☐ Behavioural & social sciences ☐ Ecological, evolutionary & environmental sciences

For a reference copy of the document with all sections, see nature.com/documents/nr-reporting-summary-flat.pdf

Life sciences study design

All studies must disclose on these points even when the disclosure is negative.

Sample size	As shown in Methods, Supplementary Methods, and Supplementary Fig. 1, 337,138 white British unrelated samples were used for the whole analyses. Randomly selected 215,768 samples were set as training sample size, 53,942 samples were set as validation dataset, 67,428 were set as 20% test dataset. 80% training and 20% validation dataset were resampled in five cross-validation manner. For non-European samples, we defined African (n=6,487), South Asian (n=7,952), and East Asian (n=1,770) individuals. We randomly split each of them into 20% validation and 80% test datasets. This represents the maximum number of unrelated individuals.
Data exclusions	For sample QC, as shown in Methods, Supplementary Methods, and Supplementary Fig. 1, we focused on unrelated individuals with genetic data based on the following criteria: (1) not reported in “Outliers for heterozygosity or missing rate” (UK Biobank Data Field 22027); (2) not reported in "Sex chromosome aneuploidy" (Data Field 22019); and (3) used to compute principal components (Data Field 22020). For non-European samples, we define African, South Asian, and East Asian samples using genotype principal components (PCs, defined in Data Field 22009) and the self-reported ancestry (Data Field 21000) as follows; African: 260 <= PC1, 50 <= PC2, and not self-identified as any of Asian, White, Mixed, or Other population groups; South Asian: 40 <= PC1 <= 120, -170 <= PC2 <= -80, and not self-identified as any of Black, White, Mixed, or Other population groups; and East Asian 130 <= PC1 <= 170, PC2 <= -230, and not self-identified as any of Black, White, Mixed, or Other population groups.<br>For SNV QC, as shown in Methods and Supplementary Fig. 1, we focused on variants passing the following criteria: (1) unambiguous single nucleotide variants (SNVs) where both reference and alternate alleles are represented by one of the four canonical nucleobases (A, T, G, C); (2) minor allele frequency (MAF) greater than 1%; (3) Hardy-Weinberg disequilibrium test p-value greater than 1.0x10-6; (4) the missingness of the variant is less than 5%; (5) imputation quality score (INFO score) greater than 0.3; and (6) present in the HapMap Phase 3 dataset. We focused on the most and the second most major alleles for multiallelic sites and set the remaining alleles as missing.
Replication	We performed five-fold cross-validation.

Randomization	Five cross-validation was conducted in random manner.
Blinding	Investigators were blind to group allocation.

Reporting for specific materials, systems and methods

We require information from authors about some types of materials, experimental systems and methods used in many studies. Here, indicate whether each material, system or method listed is relevant to your study. If you are not sure if a list item applies to your research, read the appropriate section before selecting a response.

Materials & experimental systems

n/a	Involved in the study
☒	☐ Antibodies
☒	☐ Eukaryotic cell lines
☒	☐ Palaeontology and archaeology
☒	☐ Animals and other organisms
☒	☐ Clinical data
☒	☐ Dual use research of concern
☒	☐ Plants

Methods

n/a	Involved in the study
☒	☐ ChIP-seq
☒	☐ Flow cytometry
☒	☐ MRI-based neuroimaging
